# Phyla: Towards a foundation model for phylogenetic inference

**DOI:** 10.1101/2025.01.17.633626

**Published:** 2025-01-22

**Authors:** Andrew Shen, Yasha Ektefaie, Lavik Jain, Maha Farhat, Marinka Zitnik

**Affiliations:** 1 Department of Biomedical Informatics, Harvard Medical School, Boston, MA, USA; 2 Department of Computer Science, Northwestern University, Evanston, IL, USA; 3 Harvard University, Cambridge, MA, USA; 4 Division of Pulmonary and Critical Care, Department of Medicine, Massachusetts General Hospital, Boston, MA, USA; 5 Kempner Institute for the Study of Natural and Artificial Intelligence, Harvard University, Allston, MA, USA; 6 Broad Institute of MIT and Harvard, Cambridge, MA, USA; 7 Harvard Data Science Initiative, Cambridge, MA, USA

## Abstract

Deep learning has made strides in modeling protein sequences but often struggles to generalize beyond its training distribution. Current models focus on learning individual sequences through masked language modeling, but effective protein sequence analysis demands the ability to reason across sequences, a critical step in phylogenetic analysis. Training biological foundation models explicitly for intersequence reasoning could enhance their generalizability and performance for phylogenetic inference and other tasks in computational biology. Here, we report an ongoing development of Phyla, an architecture that operates on an explicit, higher-level semantic representation of phylogenetic trees. Phyla employs a hybrid state-space transformer architecture and a novel tree loss function to achieve state-of-the-art performance on sequence reasoning benchmarks and phylogenetic tree reconstruction. To validate Phyla’s capabilities, we applied it to reconstruct the tree of life, where Phyla accurately reclassified archaeal organisms, such as Lokiarchaeota, as more closely related to bacteria—aligning with recent phylogenetic insights. Phyla represents a step toward molecular sequence reasoning, emphasizing structured reasoning over memorization and advancing protein sequence analysis and phylogenetic inference.

## 1 Introduction

Protein language models (PLM) use transformers with masked language or autoregressive self-supervision to model molecular sequences (Rives et al., 2021; Lin et al., 2022; Alley EC, 2019; Madani, 2023; Notin, 2022). PLMs have shown state-of-the-art performance across predictive (Meier et al., 2021; Rives et al., 2021; Rao et al., 2021; Elnaggar et al., 2021; Alley EC, 2019; Rao et al., 2020) and generative (Lin et al., 2022; Hayes et al., 2024; Madani, 2023; Ferruz, 2022) tasks. Despite the advantages of PLMs, they are limited in the context length of their inputs due to the quadratic nature of self-attention (Vaswani et al., 2017). State-space models have emerged as a way to increase the size of genomic context that can be integrated into a model (Gu & Dao, 2024; Poli et al., 2023; Sgarbossa et al., 2024). Hybrid architectures have emerged to combine the strength of transformers with state-space models (Nguyen et al., 2023; 2024). Despite the advances in model architecture and masked language modeling, self-supervised tasks used to train these models have remained largely the same. Recent studies have highlighted the shortcomings of masked language modeling from the lens of generalizability (Ektefaie et al., 2024), where learned representations are heavily biased by training dataset composition (Ding & Steinhardt, 2024) or do not prove useful for many tasks (Li et al., 2024), leading to worse performance of generalist foundation models than their specialized counterparts in some cases.

PLMs are trained to model individual protein sequences but are not designed to reason across sequences. As such, these models excel at capturing *intra-sequence relationships* but are not explicitly trained to handle *inter-sequence relationships*. This limitation is rooted in the architecture and training paradigm of PLMs. *Understanding evolutionary relationships requires the identification of similarities and differences across sequences before modeling finer details at the amino acid level within each sequence*. PLMs implicitly learn some degree of inter-sequence relationships through exposure to large datasets, but they lack explicit architectural or training features designed to reason systematically across sequences. We hypothesize that models with explicit hierarchical reasoning capabilities, tailored to compare and integrate information across sequences, will perform better at tasks like phylogenetic inference and functional annotation.

Important biological insights can be discerned from reasoning across sequences, whether it is understanding a taxon’s position in the tree of life (Hug, 2016), determining the impact of a protein variant (Meier et al., 2021; Brandes, 2023; et al., 2023), or annotating functions of poorly characterized proteins (Nguyen et al., 2024; Avsec, 2021; Zvyagin et al., 2022; Queen et al., 2024). PLMs using masked language tasks learn to implicitly compare sequences by observing many variations of the same sequence. However, these models tend to memorize variations rather than compare sequences, limiting their ability to generalize to unseen sequences or variants (Ektefaie et al., 2024). We hypothesize that a PLM explicitly trained to compare sequences can achieve better generalization than current models.

Assessing a model’s ability to reason across molecular sequences is challenging because the specifics of sequence comparison vary with the biological application. Biologists use multiple sequence alignment algorithms to inform phylogenetic trees. Though these algorithms are foundational tools for hypothesis generation (Chatzou et al., 2015), they are computationally intensive, with time and resource requirements that scale exponentially with sequence length and number (Katoh et al., 2002; Sievers et al., 2011). We propose that the ability to reconstruct phylogenetic trees from model-generated embeddings can serve as a proxy for evaluating sequence reasoning. Excelling at this task requires models to identify and prioritize differences between protein sequences and assess their impact on protein phylogeny. This demands the ability to reason across multiple sequences rather than modeling each sequence independently.

### Present work.

We report an ongoing development of Phyla models, an approach that shifts away from processing at the token or sequence level and moves toward hierarchical reasoning in an abstract embedding space tailored for proteins. This embedding space captures relationships and insights independent of individual sequences, focusing instead on the underlying semantic and functional connections across proteins. Phyla is a hybrid state-space and sparsified attention model trained on a novel tree loss and masked language loss. Phyla models the reasoning process at a phylogenetic and functional level, rather than being confined to individual amino acid tokens or specific sequence alignments. Phyla is explicitly trained to reconstruct the phylogenetic trees of protein sequences during training, in addition to predicting the identity of masked amino acids.

**Figure 1:**
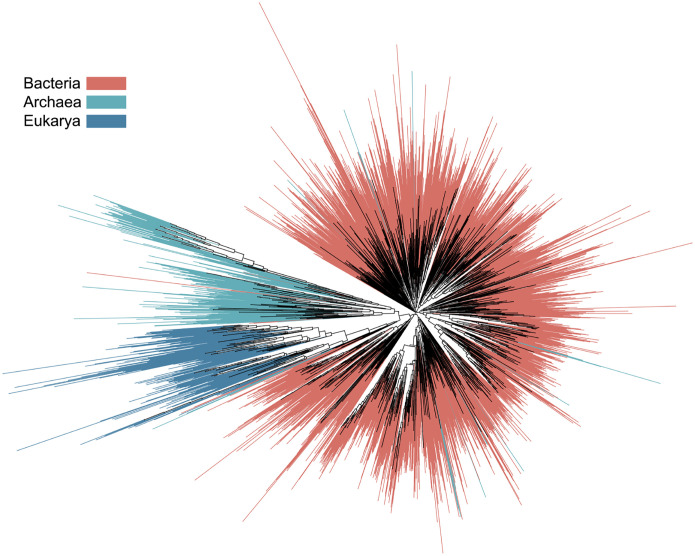
Phyla constructed phylogenetic tree of 3,084 ribosomal protein sequences among organisms in all the taxonomic domains of life. Organisms classified as bacteria are labeled in red, archaea in light blue, and eukarya in dark blue.

To evaluate Phyla’s generated trees, we introduce a sequence reasoning benchmark designed to assess the ability of PLMs to reconstruct phylogenetic trees and perform phylogenetic inference based on these trees. We find that PLMs exhibit significant limitations in their ability to reason across sequences. Phyla achieves stronger performance in phylogenetic tree reconstruction and functional prediction despite having significantly fewer parameters (291M parameters) compared to larger models. On the OpenFold small benchmark, Phyla achieves a normalized Robinson-Foulds (Robinson & Foulds, 1981) (normRF) metric of 0.8187, outperforming much larger models like ESM3 (1.4B parameters) and Evo (7B parameters). On the ProteinGym benchmark, Phyla achieves a Spearman’s rank correlation of 0.696, which is competitive with models like ESM2 (3B parameters) and better than several other methods. We use Phyla to reconstruct the tree of life from ribosomal protein sequences of eukarya, archaea, and bacteria (Figure 1). Our analysis suggests that Phyla captures relationships among subspecies of bacteria and archaea, differing from the current tree of life and aligning more closely with the functional characteristics of these subspecies. This motivates future development of Phyla as part of a new generation of sequence models aimed at advancing biological sequence reasoning.

## 2 Related Work

### Protein Language Models (PLMs).

State-of-the-art protein language models include transformer-based models such as ESM2 (Lin et al., 2022) and ProGen (Madani, 2023) that are trained using masked or autoregressive language modeling. These models learn to model the language of proteins by learning the co-occurrence of amino acid residues within a diverse training set. Other PLMs, such as ESM3 (Hayes et al., 2024), model additional data modalities. ESM3 considers structural and functional information in addition to the background amino acid sequences. These models have demonstrated good performance on intra-sequence reasoning from sequence modeling pre-training tasks but have not explicitly been trained on inter-sequence reasoning between different sequences in the training set.

### Alternatives to self-attention.

Self-attention is the backbone of the transformer but suffers from quadratic scaling with sequence length, making modeling longer protein sequences difficult (Vaswani et al. (2017)). The Mamba state-space architecture has been proposed as an alternative backbone architecture for sequence-based foundation models. The architecture builds upon the S4 class of structured state-space models (Gu et al. (2022)) by adding a selection mechanism and a hardware-aware parallel algorithm. These advances allow Mamba to model long sequences efficiently. Beyond Mamba, other approaches use similar ideas to extend context length, including Hyena (Poli et al., 2023) and xLSTM (Beck et al., 2024).

### Bioinformatics approaches to phylogenetic analysis.

Traditional tree reconstruction methods for a set of input protein sequences consist of generating a multiple sequence alignment (MSA) using one of many alignment algorithms. The MAFFT and Clustal Omega alignment algorithms are popular choices for efficient and accurate MSA generation (Katoh et al. (2002); Sievers et al. (2011)). These alignment algorithms align the input sequences by matching the location of the most conserved amino acids within the sequences. After generating the MSA, a phylogenetic tree is reconstructed using a tree reconstruction algorithm, like FastTree and IQTree (Price et al. (2010); Nguyen et al. (2014)). These algorithms infer the structure of the phylogenetic tree with and without parametric models and usually with various heuristics to generate the most likely phylogenetic tree topology. The primary limitation of tree reconstruction is runtime inefficiency as tree sizes grow.

## 3 Phyla Approach

We design and implement Phyla, which explicitly operates at two levels of abstraction: sequence tokens and phylogenetic trees. We define a phylogenetic tree as a higher-order abstraction that encapsulates evolutionary and functional relationships among sequences. This approach stands in sharp contrast to current PLMs, which are primarily sequence-centric and token-based, lacking the capacity to perform structured, multi-level reasoning across datasets. Phyla introduces a hierarchical architecture that enables reasoning in this abstract embedding space, setting a new standard for protein modeling and phylogenetic inference.

This paper aims to provide proof of concept for this high-level vision of an alternative architecture to current best practices in protein language modeling.

### 3.1 Overview of Phyla Model Architecture

Given a set of protein sequences *S*, the goal is to construct a phylogenetic tree *T* of *S*. To address this problem, we propose a hybrid state-space transformer model, Phyla. During training, a phylogenetic tree *T* is sampled, where *T* consists of *N* sequences *S*. Each sequence is tokenized into a stream of 22 tokens, corresponding to 20 standard amino acids, a mask token, and a pad token. The input to Phyla is *S* with a [*CLS*] token concatenated in front of each tokenized sequence, *s* ∈ *S*: {[*CLS*]*s*_1_ || [*CLS*]*s*_2_ || [*CLS*]*s*_3_, …, [*CLS*]*s*_*n*_} and the output is a phylogenetic tree which is then compared to the sampled tree to calculate the loss. The size and number of trees considered in each training step are determined at each training step by an adaptive batch size sampler.

The architecture of Phyla comprises of a sequence of blocks, each containing 16 Mamba layers (Gu & Dao (2024)) followed by a sparsified self-attention layer. The sparsified self-attention employs an attention mask *M*:

(1) $$M_{ij}=\{\begin{array}{l} 1,\;\text{if}\;\text{the}\;j\text{-th}\;\text{token}\;\text{is}\;\text{within}\;\text{the}\;i\text{-th}\;\text{sequence},\\ 0,\;\text{otherwise}. \end{array}$$

This architecture incorporates inductive biases tailored to sequence comparison. Specifically, Mamba layers facilitate inter-sequence comparisons, capturing relationships between different sequences, and sparsified attention layers apply self-attention between the CLS token of each input protein sequence and its sequence positions to perform intra-sequence comparisons. Phyla is trained on 13,696 phylogenetic trees from OpenProteinSet (Ahdritz et al., 2023) with 40 sequence blocks, and the current model release has 291 million model parameters.

### 3.2 Adaptive Batch Sizing

We employ an adaptive batch sizing approach to efficiently utilize GPU memory and avoid overfitting to a specific tree topology. We determine the largest subtree *t* ∈ *T* at every training step that can fit within the available GPU memory. Next, we randomly sample a subtree size *n* such that 5 ≤ *n* ≤ |*t*|, where |*t*| is the number of sequences in *t*. Finally, we identify how many subtrees of the sampled size |*t*| can be accommodated within the GPU memory. If the model encounters an out-of-memory (OOM) error during this process, the subtrees are resampled with both the subtree size and the number of subtrees halved. Details are given in Appendix A.1.

### 3.3 Phyla Loss Function

Phyla’s loss function is a combination of a masked language loss (MLM) and tree loss (TREE):

(2) $$L_{\text{PHYLA}}=L_{\text{TREE}}+L_{\text{MLM}}.$$

To compute the tree loss, we first normalize the distance matrix of the sampled tree, *D*, by dividing each element *D*_*ij*_ by the maximum value of its corresponding row *i*:

(3) $$D_{ij}^{\prime }=\frac{D_{ij}}{\text{max}\left(D_{i1},D_{i2},\ldots ,D_{iN}\right)}$$

Next, we compute the pairwise distances between embeddings of CLS tokens of the sequences to create a predicted distance matrix *P*. We then row-normalize *P* in the same way as *D*. *L*_Tree_ is the L1 loss between the row-normalized distance matrices:

(4) $$L_{\text{TREE}}=\overset{n}{\underset{i}{\sum }}\overset{n}{\underset{j}{\sum }}\left|D_{ij}^{\prime }-P_{ij}^{\prime }\right|$$

Lastly, for the MLM loss, we mask 15% of the input sequence and have the model predict the identity of the masked sequences as described previously (Devlin et al., 2019).

## 4 Experiments

### Datasets.

We evaluate the ability to reconstruct trees using a held-out subset of the OpenProteinSet (Ahdritz et al., 2024) comprising 119 trees. The trees are stratified into three categories based on the number of sequences in the tree: “Openfold Small” (0 to 1,000 sequences, 45 trees), “Openfold Medium” (1,000 to 2,000 sequences, 45 trees), and “Openfold Large” (2,000+ sequences, 29 trees). We also evaluate the ability to predict functional labels using 83 datasets from the ProteinGym (Notin et al., 2023a) benchmark. The 83 datasets were chosen based on which would fit on a single 80GB H100 GPU during inference.

### Baselines.

We consider two protein language models, one genomic foundation model, six models from the ProteinGym benchmark, and two traditional tree reconstruction methods. The protein language models include ESM2 and ESM3 (Lin et al. (2022); Hayes et al. (2024)). The genomic foundation model is Evo (Nguyen et al. (2024)). The 6 ProteinGym benchmarks include ProteinNPT, MSA Transformer, ESM-1v, Tranception, TranceptEVE, and DeepSequence (Notin et al. (2023a;b); Rao et al. (2021); Meier et al. (2021); Notin et al. (2022); Riesselman (2018); Notin (2022)). The two traditional tree reconstruction methods include MAFFT + FastTree and Clustal + FastTree (Katoh et al. (2002); Price et al. (2010); Sievers et al. (2011)).

### Evaluation setup.

We consider two evaluation settings. **Tree reconstruction**: This setting evaluates the model’s ability to reconstruct phylogenetic trees given solely the original sequences. We evaluate tree reconstruction by comparing the predicted tree to the reference tree using the Robinson-Foulds metric (Robinson & Foulds (1981)). **Functional prediction**: This setting evaluates the model’s ability to predict functional labels given solely the original sequences. We assess functional prediction by training a linear probe classifier on the generated embeddings. We also consider a case study of **reconstructing the tree of life using ribosomal protein sequences** to demonstrate a potential biological use case for Phyla.

#### 4.1 Phyla can Reason over Protein Sequences

##### Experimental setup.

To assess the ability of Phyla to reason over sequences, we assess Phyla’s ability to reconstruct phylogenetic trees on the “Openfold Small”, “Openfold Medium”, and “Openfold Large” datasets. We use the metric of Robinson-Foulds distance, or “RF”, whereby a larger RF value is equivalent to a larger distance between predicted and reference tree, and can be interpreted as a lower quality predicted tree. The RF metric is not invariant to tree size, so we compute the normalized RF, or “normRF”, to directly compare the tree reconstruction performance between trees of different sizes. We utilize the ETE3 Toolkit implementation of RF and normRF distance (Jaime Huerta-Cepas & Bork (2016)). We compare the performance of Phyla against state-of-the-art PLMs (ESM2, ESM3) and genomic foundation models (Evo) (Lin et al. (2022); Hayes et al. (2024); Nguyen et al. (2024)). Table 1 shows the normRF performance of Phyla and benchmark models on the three stratifications of the OpenProteinSet.

##### Results.

Phyla achieves the best performance on the Openfold Small evaluation set, beating benchmark models with 2 to 24 times more parameters (Table 1). Although Phyla does not outperform the benchmarks in the Openfold Medium and Openfold Large evaluation sets, these results suggest a trend in all models worsening their performance as tree size increases.

#### 4.2 Phyla Trees Encode Protein Functional Information

##### Experimental setup.

To evaluate the expressivity of the learned embeddings from Phyla, we train a linear probe on predicting functional labels from the embeddings. We utilize the 83 datasets from the ProteinGym (Notin et al. (2023a)) benchmark as our evaluation set. Table 2 shows the average Spearman correlation metric for linear probe performance on the 83 ProteinGym evaluation datasets.

##### Results.

Phyla ranks among the top 4 models out of 15 evaluated (Table 2) on the Linear Probe metric, despite having significantly fewer parameters and being trained on a smaller dataset. In contrast, Evo performs the worst among all models on this metric, which aligns with expectations given that Evo was trained on prokaryotic genomes, whereas ProteinGym comprises human protein sequences (Nguyen et al., 2024).

#### 4.3 Runtime Comparison

##### Experimental setup.

To evaluate the efficiency of Phyla compared to the benchmark models on embedding generation, we calculate the runtime required to generate sequence embeddings, generate a predicted distance matrix, and run the neighbor-joining algorithm to construct the predicted tree (scikit-bio development team, 2020). Table 3 shows the average runtime in seconds for tree reconstruction across the three stratifications of the OpenProteinSet and the ProteinGym evalution set for Phyla and benchmark models. We also include the runtime for a larger Phyla model “Phyla (660M)” with 660M parameters for a more fair comparison to the benchmark models, the smallest of which had 650M parameters.

We also compared tree reconstruction of Phyla with phylogenetic tree reconstruction methods. We calculate the runtime required to construct a multiple sequence alignment (MSA) from each dataset using the state-of-the-art aligners MAFFT and Clustal Omega, and then construct a tree from the MSA using a FastTree efficient tree construction method (Katoh et al., 2002; Price et al., 2010; Sievers et al., 2011). Table 4 shows the average runtime in seconds for tree reconstruction across the three stratifications of the OpenProteinSet for Phyla and the benchmark methods.

##### Results.

Phyla generates embeddings much faster than the benchmark models across all three stratifications of the OpenProteinSet and the ProteinGym evaluation set (Table 3). The larger “Phyla (660M)” model generates embeddings faster than all benchmarks. In addition, Phyla outperforms phylogenetic tree reconstruction methods across all stratifications of the OpenProteinSet, particularly on the Openfold Large evaluation set of trees larger than 2,000 sequences (Table 4). We see that the runtime for tree reconstruction increases as a function of tree size, but PHYLA’s absolute runtime is still significantly faster than the other methods at all scales.

#### 4.4 Ablation Analyses

##### Experimental setup.

To understand the effect of the sequence reasoning loss, we trained Phyla with only masked language modeling loss (Phyla-MLM). We evaluated (Phyla-MLM) on tree reconstruction using the three stratifications of the OpenProteinSet and also on functional prediction using the Linear Probe metric.

##### Results.

As shown in Table 5, we found Phyla-MLM consistently performed worse than Phyla on tree reconstruction and functional prediction.

#### 4.5 Using Phyla to Construct a Phylogenetic Tree Across 3,084 Organisms

Phyla demonstrates promising performance in sequence reasoning. To showcase its capabilities, we applied Phyla to the task of phylogenetic tree construction. The tree of life is a fundamental framework in biology, delineating evolutionary relationships between organisms and serving as an indicator of relative phenotypic traits. Current approaches to constructing the tree of life typically rely on multiple sequence alignments of ribosomal proteins (Hug et al., 2016). We used Phyla to analyze a set of 3,084 phylogenetic sequences, successfully reconstructing the tree of life in just 16 hours, compared to the 3,840 hours required by traditional methods (Hug et al., 2016).

**Figure 2:**
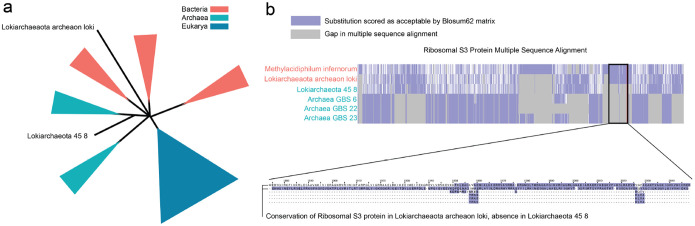
Phyla created a new placement of Lokiarchaea. a. Lokiarchaeaota archeaon loki (L-A) was placed among bacterial neighbors while Lokiarchaeota 45 8 (L-45) was placed among archaeal neighbors. b. Analysis of the multiple sequence alignment revealed that L-A placed with bacteria retained a conserved S3 ribosomal protein, aligning with its bacterial neighbors. In contrast, the L-45 placed with archaea exhibited a deletion of the S3 ribosomal protein, aligning with its archaeal neighbors.

As shown in Figure 1, Phyla accurately places sequences within their respective domains in the tree of life. Phyla identifies overlap between certain archaeal isolates and bacteria, a result consistent with current phylogenetic reasoning. Lokiarchaeota, an archaeal lineage clustered with bacteria, is known to have a mosaic genome with over 30% of its genome derived from bacteria (Levasseur et al., 2017). Within this genus, Phyla placed Lokiarchaeaota archaeon loki (L-A) paraphyletic to bacteria while Lokiarchaeota 45 8 (L-45) is paraphyletic to archaea (Figure 2a). Examination of the multiple sequence alignment of L-45 and L-A with their immediate phylgenetic neighbors, revealed that L-45 harbors a deletion of the S3 ribosomal protein while L-A retains this protein (Figure 2b). The S3 deletion has been noted in previous studies of Lokiarchaea genomes Da Cunha et al. (2017). Biologically, these differences may relate to adaptation to extreme environments. L-45 was isolated from the bottom of the Arctic Ocean, while L-A was isolated from the Horonobe Underground Research Laboratory (URL) in Japan. In fact, L-A’s neighbor, Methylacidiphilum infernorum, is an acidophilic methanotroph originally isolated from a geothermal area in New Zealand Hou et al. (2008). This environment shares similarities with the conditions in the URL, where extensive methane metabolism has been observed Amano et al. (2024). This highlights PHYLA’s ability to discover potentially biologically meaningful evolutionary relationships.

## 5 Conclusion

Molecular sequence reasoning presents unique challenges, requiring models to represent individual sequences while reasoning across multiple sequences at varying levels of abstraction. Here, we report an ongoing development of Phyla, a hybrid state-space and transformer model that operates at two levels of abstraction: sequence tokens and phylogenetic trees. By defining phylogenetic trees as higher-order abstractions that encapsulate evolutionary and functional relationships among sequences, Phyla can overcome limitations of current protein language models, which are primarily sequence-centric and token-based. This hierarchical architecture enables structured, multi-level reasoning and sets a new benchmark for molecular sequence modeling and phylogenetic inference.

Preliminary results show that Phyla achieves competitive or state-of-the-art performance in reconstructing phylogenetic trees, outperforming traditional multiple sequence alignment algorithms and existing machine learning approaches in runtime efficiency. Using Phyla, we reconstructed the tree of life, revealing a phylogeny that aligns with established biological reasoning. These results motivate future development of Phyla to establish a foundational model for molecular sequence reasoning and more efficient and insightful phylogenetic analysis.

## Supplementary Material

1

## Figures and Tables

**Table 1:** Tree reconstruction performance. Average normRF metric values (lower value indicates better performance) across all datasets within the Openfold Small (0 to 1,000 sequences), Medium (1,000 to 2,000 sequences), and Large (2,000+ sequences) evaluation sets for Phyla vs. ESM models vs. Evo.

Model	Openfold Small normRF ↓	Openfold Medium normRF↓	Openfold Large normRF ↓
ESM2 (650M)	0.8735	0.9084	0.9292
ESM2 (3B)	0.8391	**0.8609**	**0.8859**
ESM3 (1.4B)	0.9070	0.9297	0.9387
Evo (7B)	0.9877	0.9949	0.9963
Phyla (291M)	***0.8187***	*0.8980*	*0.9357*

**Table 2:** Functional prediction performance. Average Spearman correlation coefficient values (higher values indicate better performance) averaged across 83 datasets within the ProteinGym evaluation sets using Linear Probe calculation for Phyla vs. ESM models vs. Evo vs. ProteinGym benchmarks, *: pulled from pre-computed ProteinGym benchmark. Note that Evo model is trained on millions of microbial genomes and thus is not expected to generalize well to human protein sequences in ProteinGym.

Model	ProteinGym Spearman correlation ↑
ESM2 (650M)	**0.7754**
ProteinNPT*	0.7081
ESM2 (3B)	0.7044
Phyla (291M)	*0.6962*
MSA Transformer Embeddings*	0.6944
ESM-1v Embeddings*	0.6482
Tranception Embeddings*	0.6239
TranceptEVE + One-Hot Encodings*	0.4839
MSA Transformer + One-Hot Encodings*	0.4738
Tranception + One-Hot Encodings*	0.4672
DeepSequence + One-Hot Encodings*	0.4591
ESM-1v + One-Hot Encodings*	0.4415
ESM3 (1.4B)	0.2743
One-Hot Encodings*	0.2725
Evo (7B)	−0.0044

**Table 3:** Runtime analyses. Average runtime in seconds (lower is better) averaged across all datasets within the Openfold Small (0 to 1,000 sequences), Medium (1,000 to 2,000 sequences), Large (2,000+ sequences), and ProteinGym evaluation sets for Phyla vs. ESM models vs. Evo.

Model	Openfold Small Seconds ↓	Openfold Medium Seconds ↓	Openfold Large Seconds ↓	ProteinGym Seconds ↓
ESM2 (650M)	12.65	58.17	280.15	78.49
ESM2 (3B)	37.96	138.22	509.97	93.14
ESM3 (1.4B)	18.38	70.18	251.55	110.73
Evo (7B)	37.02	126.47	477.72	78.38
Phyla (291M)	***2.08***	***23.38***	***179.00***	***69.74***
Phyla (660M)	***3.22***	***26.59***	***216.17***	***70.52***

**Table 4:** Runtime analyses. Average runtime in seconds (lower is better) averaged across all datasets within the Openfold Small (0 to 1,000 sequences), Medium (1,000 to 2,000 sequences), Large (2,000+ sequences), and ProteinGym evaluation sets for Phyla vs. traditional benchmarks that involve performing multiple-sequence alignment followed by running a phylogenetic tree construction algorithm on the aligned sequences.

Model	Openfold Small Seconds ↓	Openfold Medium Seconds ↓	Openfold Large Seconds ↓
MAFFT + FastTree	38.20	190.02	695.67
Clustal + FastTree	26.71	354.53	1594.88
Phyla (291M)	***2.08***	***23.38***	***179.00***

**Table 5:** Tree loss ablation performance. Average normRF metric values (lower is better) across all datasets within the Openfold Small (0 to 1,000 sequences), Medium (1,000 to 2,000 sequences), and Large (2,000+ sequences) evaluation sets and average Spearman rank correlation (higher is better) averaged across 83 datasets within the ProteinGym evaluation set for Phyla vs. Phyla-MLM.

Model	Openfold Small normRF ↓	Openfold Medium normRF ↓	Openfold Large normRF ↓	ProteinGym Spearman ↑
Phyla-MLM	0.9306	0.9663	0.9711	0.6174
Phyla	***0.8187***	***0.8980***	***0.9357***	**0.6962**

## Data Availability

Data to run Phyla can be found in the project github specifically in the ”dataset_info” folder.

## A Appendix

### A.1 Adaptive batch size algorithm

We empirically determined that our model could process inputs of length 213,350 on a 32 GB GPU and 302,350 on a 48 GB GPU. For untested GPU memory sizes, we used a linear model to estimate the maximum input length. Given the length of the longest sequence in a phylogenetic tree, we calculated the largest tree that can fit within the available GPU memory. To mitigate overfitting, we randomly sampled a tree size between 5 and the maximum permissible tree size. From the sampled tree size, we determined the number of trees we could sample.

If an out-of-memory (OOM) error occurred during training, the model resampled with both the tree size and the number of trees halved.

### A.2 Phylogenetic Tree Reconstruction

See Figure S1 and Figure S2.

### A.3 Functional Prediction

See Figure S3 and Figure S4.

### A.4 Runtime

See Figure S5 and Figure S6.

### A.5 Model Size

Phyla has 291M model parameters, which is less than 650M parameters of ESM2, 1.4B parameters of ESM3, 3B parameters or large ESM2, and 7B parameters of Evo model. We also train on 4 GPUs for 6 days, which is less than the 512 GPUs that ESM2 trains on for 8 days. We also train on a much smaller training set of ~13,000 sequences compared to the ~50,000,000 sequences that ESM2 trains on.

### A.6 Ablations

Tree loss ablation results in worse performance on tree reconstruction and functional prediction (for linear probe performance). See Figure S7 and Figure S8.
