# Sequence Modeling Is Not Evolutionary Reasoning

**DOI:** 10.1101/2025.01.17.633626

**Published:** 2025-06-27

**Authors:** Yasha Ektefaie, Andrew Shen, Lavik Jain, Maha Farhat, Marinka Zitnik

**Affiliations:** Department of Biomedical Informatics, Harvard Medical School, Boston, MA 02115; Department of Biomedical Informatics, Harvard Medical School, Boston, MA 02115; Department of Biomedical Informatics, Harvard Medical School, Boston, MA 02115; Department of Biomedical Informatics, Harvard Medical School, Boston, MA 02115; Department of Biomedical Informatics, Harvard Medical School, Boston, MA 02115

## Abstract

Protein language models (PLMs) are commonly assumed to capture evolutionary information by training on large protein sequence datasets. However, it remains unclear whether PLMs can reason about evolution—that is, infer evolutionary relationships between protein sequences. To test this capability, we introduce a benchmark for evolutionary reasoning and find that existing PLMs consistently fail to recover phylogenetic structure, despite strong performance on standard tasks such as masked token prediction and contact prediction. To address this limitation, we present Phyla. Phyla introduces a hybrid state-space and transformer architecture that jointly process multiple sequences and is trained using a tree-based objective over 3,000 phylogenies spanning diverse protein families. Phyla achieves state-of-the-art performance in evolutionary reasoning, outperforming the next-best model by 13% on tree reconstruction and 10% on taxonomic clustering. Beyond synthetic benchmarks, Phyla applies to real-world settings: it reconstructs biologically accurate branches of the tree of life and infers whole-genome evolutionary relationships from *Mycobacterium tuberculosis* genomes. These findings suggest that evolutionary reasoning is not an emergent property of large-scale sequence modeling. Instead, Phyla shows that models trained with phylogenetic supervision can reason about evolution more effectively, offering a biologically grounded path toward evolutionary foundation models.

## 1 Introduction

Protein language models (PLMs) use transformers with masked language or autoregressive self-supervision to model molecular sequences (Rives et al., 2021; Lin et al., 2022; Alley EC, 2019; Madani, 2023; Notin, 2022). PLMs have shown state-of-the-art performance across predictive (Meier et al., 2021; Rives et al., 2021; Rao et al., 2021; Elnaggar et al., 2021; Alley EC, 2019; Rao et al., 2020) and generative (Lin et al., 2022; Hayes et al., 2024; Madani, 2023; Ferruz, 2022) tasks. At their core, PLMs learn statistical distributions over amino acid sequences, modeling which residues are likely to appear given their surrounding context. This enables powerful zero-shot predictions of mutational effects by observing how the likelihood of a substitution changes under the model. Over time, this paradigm has been framed as a form of “evolutionary modeling,” with the assumption that capturing residue-level distributions implicitly reflects evolutionary dynamics.

However, in this work, we argue that this paradigm conflates *evolutionary modeling with evolutionary reasoning*. Evolutionary modeling, as practiced by current PLMs, involves capturing local amino acid distributions to estimate residue likelihoods. In contrast, evolutionary reasoning requires a deeper level of abstraction: given a set of sequences, can a model infer the evolutionary relationships that generated them? This includes identifying shared ancestry, relative divergence, and tree-like structure — tasks that cannot be solved by distributional modeling alone.

This distinction is critical. Evolutionary reasoning is the foundation of phylogenetic analysis — a problem that predates modern biology and underlies centuries of effort to reconstruct how life has changed through time. Classical phylogenetics attempts to recover the tree of life Hug (2016) from observed sequences, but current PLMs are not evaluated on their ability to do this. They operate largely in isolation, modeling individual sequences without explicit multi-sequence comparison, structure discovery, or relational inference. Beyond phylogeny, important biological insights can be discerned from reasoning across sequences, whether it is determining the impact of a protein variant (Meier et al., 2021; Brandes, 2023; et al., 2023) or annotating functions of poorly characterized proteins (Nguyen et al., 2024; Avsec, 2021; Zvyagin et al., 2022; Queen et al., 2024)

This work poses the following question: *Are current PLMs capable of evolutionary reasoning? And if not, how must we reimagine their architecture and training to enable them to do so?*

### Present Work.

To address the limitations of current PLMs in performing evolutionary reasoning, we make several contributions. First, we introduce the first benchmark for evolutionary reasoning over protein sequences, built from curated datasets containing ground-truth phylogenetic trees derived from established phylogenetic studies and databases. This benchmark enables rigorous evaluation of whether models can infer evolutionary structure from sets of sequences, rather than simply modeling local residue distributions. Second, we evaluate existing PLMs on this benchmark. Using their learned embeddings, we attempt to reconstruct phylogenetic trees and find that these models Lin et al. (2022); Hayes et al. (2024); ESM Team (2024); Nijkamp et al. (2022); Brixi et al. (2025) fail to outperform a simple baseline: Hamming distance. This analysis highlights a fundamental limitation: current PLMs are architecturally and functionally optimized for intra-sequence modeling, not inter-sequence reasoning.

To address this gap, we propose Phyla, a new model architecture designed for evolutionary reasoning. Phyla is a hybrid state-space and sparsified attention model that processes multiple sequences jointly. It alternates between inter-sequence modules, which detect conserved motifs across sequences, and intra-sequence modules, which contextualize these motifs within individual sequences—allowing the model to reason over sequence sets. We also introduce a novel pretraining objective tailored to phylogenetic structure. Instead of using masked language modeling, Phyla is trained using a tree loss that teaches the model to embed sequences such that their pairwise distances reflect the correct evolutionary topology.

Despite its compact size (24M parameters), Phyla achieves state-of-the-art performance in phylogenetic tree reconstruction and competitive accuracy in mutation effect prediction. On TreeBase—a dataset of protein sequences paired with phylogenetic trees from published studies—Phyla outperforms much larger models, including ESM-3 (1.4B) and ProGen-XLarge (6.4B), by 8.5% and 15.7% respectively, based on normalized Robinson-Foulds (normRF) distance. On the ProteinGym mutation effect prediction benchmark, it achieves a Spearman correlation of 0.6380, matching or exceeding models with several orders of magnitude more parameters. We further demonstrate Phyla’s capacity for evolutionary reasoning by applying it to reconstruct the tree of life from ribosomal protein sequences spanning bacteria, archaea, and eukarya. Additionally, we use Phyla to infer whole-genome evolutionary relationships among Mycobacterium tuberculosis isolates. In both cases, the resulting trees deviate from canonical topologies and instead reflect known functional distinctions, suggesting that Phyla learns representations aligned with true biological structure. These results position Phyla as a foundation for a new generation of protein models grounded in evolutionary reasoning rather than sequence-level pattern recognition alone.

## 2 Related Work

### Protein Language Models (PLMs).

State-of-the-art protein language models include transformer-based models such as ESM2 (Lin et al., 2022), ESMC ESM Team (2024), ProGen (Madani, 2023), and Progen2 Nijkamp et al. (2022) that are trained using masked or autoregressive language modeling. These models learn to model the language of proteins by learning the co-occurrence of amino acid residues within a diverse training set. Other PLMs, such as ESM3 (Hayes et al., 2024), model additional data modalities. ESM3 considers structural and functional information in addition to the background amino acid sequences. These models have demonstrated good performance on intra-sequence reasoning from sequence modeling pre-training tasks but have not explicitly been trained on inter-sequence reasoning between different sequences in the training set.

### Alternatives to self-attention.

Self-attention is the backbone of the transformer but suffers from quadratic scaling with sequence length, making modeling longer protein sequences difficult (Vaswani et al. (2017)). The Mamba state-space architecture has been proposed as an alternative backbone architecture for sequence-based foundation models. The architecture builds upon the S4 class of structured state-space models (Gu et al. (2022)) by adding a selection mechanism and a hardware-aware parallel algorithm. These advances allow Mamba to model long sequences efficiently. Beyond Mamba, other approaches use similar ideas to extend context length, including Hyena (Poli et al., 2023a) and xLSTM (Beck et al., 2024). More recent work has shown hybrid transformer and attention architectures allow for long-context modeling while maintaining the advantages of transformers Poli et al. (2023b); Ren et al. (2025).

### Bioinformatics approaches to phylogenetic analysis.

Traditional tree reconstruction methods for a set of input protein sequences consist of generating a multiple sequence alignment (MSA) using one of many alignment algorithms. The MAFFT and Clustal Omega alignment algorithms are popular choices for efficient and accurate MSA generation (Katoh et al. (2002); Sievers et al. (2011)). These alignment algorithms align the input sequences by matching the location of the most conserved amino acids within the sequences. After generating the MSA, a phylogenetic tree is reconstructed using a tree reconstruction algorithm, like FastTree and IQTree (Price et al. (2010); Nguyen et al. (2014)). These algorithms infer the structure of the phylogenetic tree with and without parametric models and usually with various heuristics to generate the most likely phylogenetic tree topology. The primary limitation of tree reconstruction is runtime inefficiency as tree sizes grow. More recent works can be found in the Appendix A.7.

## 3 Problem Definition

### Notation

We denote a collection of n protein sequences as $S=\left\{s_{1},s_{2},\ldots ,s_{n}\right\}$, where each $s_{i}$ is a variable-length sequence over the alphabet Σ of 20 standard amino acids: Σ={A,C,…,Y}. A phylogenetic tree is denoted by T, with leaves in bijection with S, and subtrees as t∈T. The true evolutionary distance between two sequences is denoted $d_{\text{evol}}(i,j)$, and we define $D_{\text{true}}\in R^{n\times n}$ and $D_{\text{pred}}\in R^{n\times n}$ as the ground-truth and predicted distance matrices, respectively.

Our model Phyla learns a parametric encoder $f_{\theta }\;:\;\Sigma^{*}\to R^{d}$ that maps each sequence $s_{i}$ to an embedding $f_{\theta }\left(s_{i}\right)$. Predicted distances are computed as:

$$D_{ij}=\left\|f_{\theta }\left(s_{i}\right)-f_{\theta }\left(s_{j}\right)\right\|.$$

We define quartets as 4-tuples of distinct indices (i,j,k,ℓ) drawn from {1,…,n}, and let 𝒬 be the set of sampled quartets. Each quartet defines three possible unrooted tree topologies: ij|kℓ,ik|jℓ, and iℓ∣jk. The temperature parameter $T^{*}$ controls the sharpness of the softmax logits in quartet loss.

### Problem

Given a collection of variable-length protein sequences $S=\left\{s_{1},\ldots ,s_{n}\right\}$, our goal is to reconstruct the latent evolutionary history that generated them, represented as a strictly binary phylogenetic tree T whose leaves correspond to the sequences in S.

To achieve this, we learn a distance-preserving encoder $f_{\theta }$ such that the embedding-based distance $D_{ij}=\left\|f_{\theta }\left(s_{i}\right)-f_{\theta }\left(s_{j}\right)\right\|$ approximates the true evolutionary distance $d_{\text{evol}}(i,j)$. We then reconstruct T by applying a fast distance-based tree builder to the predicted matrix $D_{\text{pred}}$.

### Alignment-Free and Guide-Tree-Free

Unlike most prior methods, our approach does not rely on a multiple sequence alignment (MSA) or a predefined guide tree (i.e., a fixed topology to scaffold inference). Removing both introduces new challenges: alignment and tree inference are jointly NP-hard WANG & JIANG (1994); Roch (2005), and guide trees offer strong structural priors. By dispensing with both, Phyla must discover meaningful patterns directly from unaligned sequences—capturing evolutionary structure without relying on handcrafted heuristics.

## 4 Methods

### 4.1 Overview of Phyla Model Architecture

To do well on evolutionary reasoning, models must be able to perform good inter- and **intra-sequence reasoning**. Inter-sequence reasoning requires the model to detect motifs that recur across different sequences, revealing shared evolutionary signatures. **Intra-sequence reasoning** requires the model to contextualize identified motifs within the context of an individual sequence to determine evolutionary divergence from the identified motif.

To meet these requirements, we introduce a hybrid state-space transformer model, Phyla. Phyla alternates between inter-sequence and intra-sequence reasoning blocks. Inter-sequence blocks employ a parameter-efficient state-space module with gated linear recurrence and deep receptive fields, enabling the model to detect long-range evolutionary motifs across sequences. Intra-sequence blocks then use sparsified attention to contextualize these motifs within each sequence (Appendix A.1.1).

Unlike most PLMs which operate on a single sequence at a time (Lin et al. (2022); Hayes et al. (2024); ESM Team (2024); Notin et al. (2023b); Nijkamp et al. (2022)), **Phyla is the first PLM designed to process multiple sequences concurrently**. This enables it to directly compare sequences, identify conserved motifs, and exploit these inter-sequence relationships to infer evolutionary structure at inference time. While prior models have employed hybrid state-space transformer architectures (Poli et al. (2023b)), Phyla introduces a sparsification mechanism: an attention mask M that constrains each token to attend only to tokens within its own sequence, preserving intra-sequence context while enabling efficient multi-sequence processing. Specifically M:

(1) $$M_{ij}=\left\{\begin{array}{ll} 1, & \text{if the}\;j\text{-th token is within the}\;i\text{-th sequence,}\\ 0, & \text{otherwise.} \end{array}\right.$$

### 4.2 Phyla Model Training

#### Tree Loss Pretraining.

To teach Phyla how to model input sequences to recapitulate evolutionary relationships, we designed a novel tree loss pretraining function. Constructing a phylogenetic tree contains a series of decisions in how to split sequences based on their relative distance to each other. With this in mind, we use a *quartet loss* to supervise training. The quartet loss encourages the model to embed sequences such that inferred distances preserve correct quartet topologies. For a set of n sequences with predicted pairwise distances $D_{\text{pred}}\in R^{n\times n}$ and true distances $D_{\text{true}}\in R^{n\times n}$, we sample a set of quartets 𝒬={(i,j,k,ℓ)}. For each quartet, we compute three possible pairwise distance sums:

$$s_{ij|k\ell }=D_{ij}+D_{k\ell },$$

$$s_{ik|j\ell }=D_{ik}+D_{j\ell ,}$$

$$s_{i\ell |jk}=D_{i\ell }+D_{jk,}$$

and convert them into logits using a softmax-like transformation:

$${\text{logits}}_{q}=\frac{1}{T^{*}}\left(\left[\begin{array}{lll} s_{ij|k\ell } & s_{ik|j\ell } & s_{i\ell |jk} \end{array}\right]-\text{mean}\left(s_{ij|k\ell },s_{ik|j\ell },s_{i\ell |jk}\right)\right),$$

where $T^{*}$ is a temperature hyperparameter controlling softness. We compute the cross-entropy loss between these logits and the ground-truth minimum sum (from $D_{\text{true}}$):

$${ℒ}_{\text{quartet}}=\frac{1}{\left|𝒬\right|}\sum_{q\in 𝒬}\text{CrossEntropy}\left({\text{logits}}_{q},\text{arg}\underset{\alpha \in \left\{ij\left|k\ell ,ik\right|j\ell ,i\ell |jk\right\}}{\text{min}}s_{\alpha }^{\text{true}}\right).$$

Quartet loss is the sole pretraining objective used to train Phyla. **In contrast to most protein language models, which rely on masked language modeling (MLM) over amino acids, Phyla does not use MLM**.

#### Training Phyla.

During training, a phylogenetic tree T is sampled, where T consists of N sequences S. Each sequence is tokenized with an alphabet 23 tokens, corresponding to 20 standard amino acids, a CLS token, a mask token, and a pad token. The input to Phyla is S with a [CLS] token concatenated in front of each tokenized sequence, $s\in S:\;\left\{\left[CLS\right]s_{1}\left\|\left[CLS\right]s_{2}\right\|\right.\left.[CLS]s_{3},\ldots ,[CLS]s_{n}\right\}$. The size and number of trees considered in each training step are determined at each training step by an adaptive batch size sampler.

We employ an adaptive batch sizing approach to efficiently utilize GPU memory and avoid overfitting to a specific tree topology. We determine the largest subtree t∈T at every training step that can fit within the available GPU memory. Next, we randomly sample a subtree size n such that 10≤n≤|t|, where |t| is the number of sequences in t. Finally, we identify how many subtrees of the sampled size |t| can be accommodated within the GPU memory. If the model encounters an out-of-memory (OOM) error during this process, the subtrees are resampled with both the subtree size and the number of subtrees halved. Further implementation details are provided in the Appendix A.1.2.

## 5 Experiments

### Datasets.

We evaluate Phyla on a range of evolutionary and functional reasoning tasks. For phylogenetic tree reconstruction, we use two held-out datasets: TreeBase, which includes 1,533 curated phylogenetic trees across diverse species (Piel & Tannen (2009)), and TreeFam, which contains 9,586 gene-family trees spanning a wide evolutionary range (Li et al. (2006)). Dataset statistics are provided in Table 3 and Table 4, with further details in Appendix A.2.1. To assess taxonomic classification, we use bacterial isolate sequences from the Genome Taxonomy Database (GTDB) (Parks et al. (2021)). We define five classification tasks at different levels of taxonomic granularity: Class, Order, Family, Genus, and Species. These tasks measure the model’s ability to cluster sequences according to hierarchical evolutionary relationships. Together, these seven tasks—tree reconstruction in TreeBase and TreeFam, and taxonomic classification at five levels using GTDB—form our evolutionary reasoning benchmark. To evaluate performance beyond evolutionary structure, we also assess performance on functional prediction using the ProteinGym benchmark, which consists of 83 protein mutation effect datasets (Notin et al., 2023a) (Appendix A.2.2).

### Baselines.

We consider four protein language models, one genomic foundation model, six models from the ProteinGym benchmark, and a naive Hamming distance baseline. The protein language models include ESM2, ESM3, ESM C, and ProGen2 (Lin et al. (2022); Hayes et al. (2024); ESM Team (2024); Nijkamp et al. (2022)). The genomic foundation model is Evo 2 (Brixi et al. (2025)). The six models from the ProteinGym benchmark are ProteinNPT, MSA Transformer, ESM-1v, Tranception, TranceptEVE, and DeepSequence (Notin et al. (2023a,b); Rao et al. (2021); Meier et al. (2021); Notin et al. (2022); Riesselman (2018); Notin (2022)).

### Evaluation setup.

We consider three evaluation settings. **Tree reconstruction**: This setting evaluates the model’s ability to reconstruct phylogenetic trees given solely the original sequences. We evaluate tree reconstruction by comparing the predicted tree to the reference tree using the normalized Robinson-Foulds metric (Robinson & Foulds (1981)). **Taxonomic clustering**: This setting evaluates the model’s ability to perform fine-grained and coarse-grained classification of sequences. We cluster 500 sequences at each taxonomic level of Class, Order, Family, Genus, and Species and assess the homogeneity of the predicted clusters. **Functional prediction**: This setting evaluates the model’s ability to predict functional labels given solely the original sequences. We assess functional prediction by training a linear probe classifier on the generated embeddings, and also by utilizing the predicted tree structure to assign labels. More details can be found in Appendix A.2.1.

### 5.1 Phyla can Reason over Protein Sequences

#### Experimental setup.

To assess the ability of Phyla to reason over sequences, we assess Phyla’s ability to reconstruct phylogenetic trees on the TreeBase and TreeFam datasets. We use the metric of Robinson-Foulds distance, or “RF”, whereby a larger RF value is equivalent to a larger distance between predicted and reference tree, and can be interpreted as a lower quality predicted tree. The RF metric is not invariant to tree size, so we compute the normalized RF, or “normRF”, to directly compare the tree reconstruction performance between trees of different sizes. We utilize the ETE3 Toolkit implementation of RF and normRF distance (Jaime Huerta-Cepas & Bork (2016)). In addition, we assess Phyla’s ability to cluster sequences across various taxonomic levels. We report the metric of homogeneity across predicted clusters as a measure of the ability to classify sequences. We compare the performance of Phyla against PLMs (ESM2, ESM3, ESM C, ProGen2) and a genomic foundation model (Evo 2) (Lin et al. (2022); Hayes et al. (2024); ESM Team (2024); Nijkamp et al. (2022); Brixi et al. (2025)). Table 1 shows the normRF performance of Phyla and benchmark models on the TreeBase and TreeFam datasets.

#### Results.

Phyla achieves the best performance on both the TreeBase and TreeFam tree reconstruction benchmarks, outperforming all tested baselines—including models with 12 to 266 times more parameters, such as ESM2 (650M) and ProGen2-XLarge (6.4B) (Table 1). On TreeFam, Phyla reduces normRF by 13.4% compared to the next-best model (ESM2 650M). On the taxonomy clustering benchmark, Phyla also achieves the best performance across all taxonomic levels, improving species-level homogeneity by 23.2% over the strongest baseline. Notably, all PLMs underperform compared to the naive Hamming distance baseline on TreeBase, highlighting the challenge of this dataset. These results underscore the effectiveness of incorporating evolutionary structure into Phyla architecture and training.

### 5.2 Phyla Trees Encode Protein Functional Information

#### Experimental setup.

To evaluate the expressivity of the learned embeddings from Phyla, we train a linear probe on predicting functional labels from the embeddings for the baseline methods. We also integrate functional annotations with the constructed trees and utilize the tree structures to infer phenotypic labels to evaluate whether the trees generated by Phyla encode meaningful biological information. To generate functional predictions from tree structure, we overlay functional labels from the train sequences onto the tree. Next, local sequence clusters are located using TreeCluster (Balaban M (2019)), which assigns cluster membership to each sequence in the evaluation set. Finally, the predicted functional label for a test sample within a certain cluster is assigned by calculating the average of the training sample labels within that cluster. We then calculate the spearman rank correlation between predicted and real values as the final metric. We utilize the 83 datasets from the ProteinGym (Notin et al. (2023a)) benchmark as our evaluation set (Appendix A.2.2). Figure 3 shows the average Spearman correlation metric with both the dataset size (in number of sequences trained on) and the model size (in parameters) on the 83 ProteinGym evaluation datasets.

#### Results.

Phyla achieves competitive performance on the ProteinGym benchmark, ranking among the top-performing models across the 83 functional prediction tasks (Figure 3). Despite being trained on only 34 times less data and with 27 times fewer parameters than models like ESM2 (650M), Phyla achieves a comparable Spearman correlation of 0.638. Notably, Phyla outperforms several larger PLMs, including ESM3 (1.4B) and Tranception, demonstrating that its tree-based representations encode meaningful biological function. These results suggest that the structured embeddings produced by Phyla not only capture phylogenetic and taxonomic information but also generalize to downstream functional prediction—despite minimal model size and data requirements.

### 5.3 Ablation Analyses

#### Experimental setup.

To understand the effect of the tree loss versus traditional masked langauge modeling loss, we trained Phyla with only masked language modeling loss (Phyla-MLM). To probe the effect of Phyla architecture on performance, we ablated the sparsification attention layers, the intra-sequence reasoning blocks (Phyla-NoAttention). We then evaluated both ablated Phyla models on the tree reasoning benchmark and on functional prediction.

#### Results.

As shown in Table 2, pretraining on a masked language modeling (MLM) objective resulted in substantial performance degradation across all tasks: a 19.6% increase in normalized Robinson-Foulds (normRF) distance for tree reconstruction (TreeBase), a 13.2% drop in Spearman correlation for functional prediction (ProteinGym), and a 5.5% reduction in average taxonomic clustering homogeneity. Similarly, ablating sparsified attention led to even greater degradation: a 20.6% increase in normRF, a 31.4% drop in functional prediction, and a 7.3% reduction in taxonomic clustering. These results highlight that both the tree-based training objective and the sparsified attention mechanism are critical to Phyla’s overall performance. In contrast, relying solely on MLM pretraining without architectural or objective innovations significantly harms phylogenetic, taxonomic, and functional understanding.

### 5.4 Phyla application to real world datasets

Phyla demonstrates promising performance in sequence reasoning. To showcase its capabilities, we applied Phyla to the task of phylogenetic tree construction. The tree of life is a fundamental framework in biology, delineating evolutionary relationships between organisms and serving as an indicator of relative phenotypic traits. Current approaches to constructing the tree of life typically rely on multiple sequence alignments of ribosomal proteins (Hug et al., 2016). We used Phyla to analyze a set of 3,084 phylogenetic sequences, successfully reconstructing the tree of life in just 16 hours, compared to the 3,840 hours required by traditional methods (Hug et al., 2016) (Appendix A.3).

**Figure 1:**
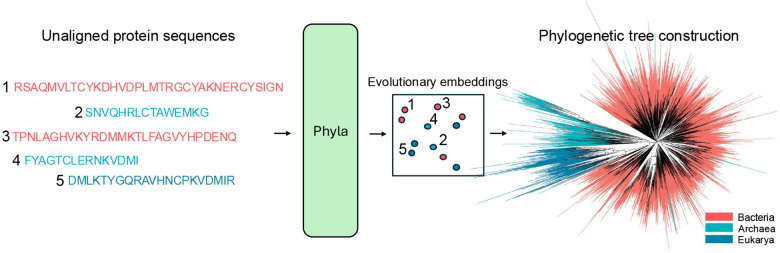
Phyla takes unaligned protein sequences and generates embeddings that reflects relative evolutionary distance between input sequences which we can use to construct phylogenetic trees.

**Figure 3:**
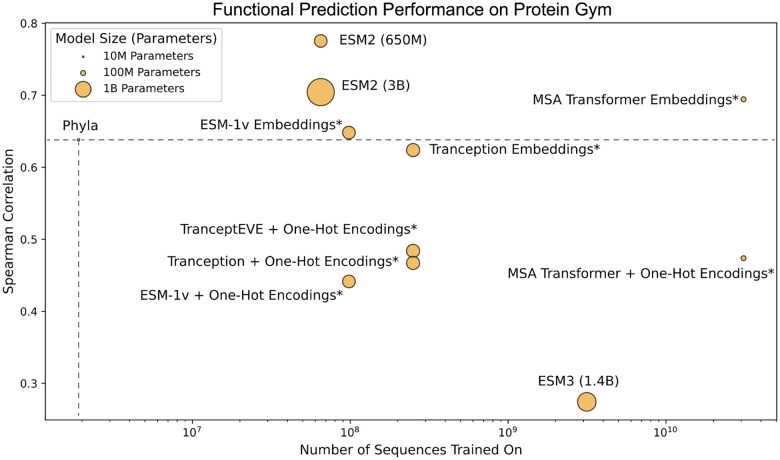
Functional prediction performance on ProteinGym with model sizes and dataset sizes. Spearman correlation of existing PLMs and Phyla on ProteinGym benchmark as a function of pretraining dataset size. Model points are sized based on the number of parameters in the model.

**Figure 4:**
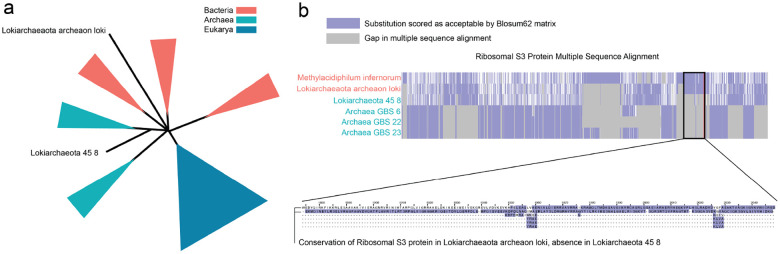
Phyla created a new placement of Lokiarchaea. a. Lokiarchaeaota archeaon loki (L-A) was placed among bacterial neighbors while Lokiarchaeota 45 8 (L-45) was placed among archaeal neighbors. b. Analysis of the multiple sequence alignment revealed that L-A placed with bacteria retained a conserved S3 ribosomal protein, aligning with its bacterial neighbors. In contrast, the L-45 placed with archaea exhibited a deletion of the S3 ribosomal protein, aligning with its archaeal neighbors.

As shown in Figure 1, Phyla accurately places sequences within their respective domains in the tree of life. Phyla identifies overlap between certain archaeal isolates and bacteria, a result consistent with current phylogenetic reasoning. Lokiarchaeota, an archaeal lineage clustered with bacteria, is known to have a mosaic genome with over 30% of its genome derived from bacteria (Levasseur et al., 2017). Within this genus, Phyla placed Lokiarchaeaota archaeon loki (L-A) paraphyletic to bacteria while Lokiarchaeota 45 8 (L-45) is paraphyletic to archaea (Figure 4a). Examination of the multiple sequence alignment of L-45 and L-A with their immediate phylgenetic neighbors, revealed that L-45 harbors a deletion of the S3 ribosomal protein while L-A retains this protein (Figure 4b). The S3 deletion has been noted in previous studies of Lokiarchaea genomes Da Cunha et al. (2017). Biologically, these differences may relate to adaptation to extreme environments. L-45 was isolated from the bottom of the Arctic Ocean, while L-A was isolated from the Horonobe Underground Research Laboratory (URL) in Japan. In fact, L-A’s neighbor, Methylacidiphilum infernorum, is an acidophilic methanotroph originally isolated from a geothermal area in New Zealand Hou et al. (2008). This environment shares similarities with the conditions in the URL, where extensive methane metabolism has been observed Amano et al. (2024). This highlights Phyla’s ability to discover potentially biologically meaningful evolutionary relationships. We also apply Phyla to whole-genome sequences of Mycobacterium tuberculosis, aligning over 4 megabases of DNA to infer novel phylogenetic relationships among clinical isolates (Appendix A.4).

## 6 Conclusion

We introduced an evolutionary reasoning benchmark and showed that existing protein language models (PLMs) struggle to capture evolutionary structure, indicating their latent spaces do not reflect phylogenetic relationships among sequences. To address this gap, we proposed Phyla, a hybrid state-space transformer trained with a novel tree-based loss function. Phyla achieves state-of-the-art performance on the evolutionary reasoning benchmark, while also performing competitively on functional prediction tasks—despite being trained on 34 times less data and with 27 times fewer parameters than models like ESM2 650M. Ablation studies confirmed that evolutionary reasoning does not emerge from sequence modeling alone and requires explicit inductive bias. Limitations and future directions are discussed in Appendix A.8.

## Supplementary Material

1

## Figures and Tables

**Figure 2:**
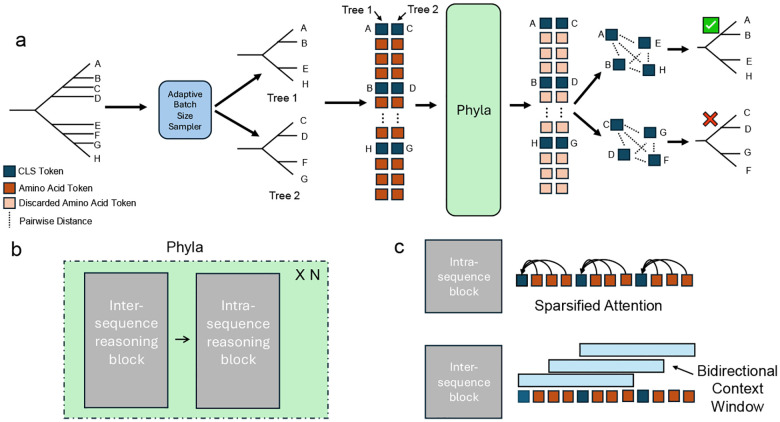
Overview of the Phyla. (a) Phyla processes batches of protein sequences sampled from a tree, learning embeddings used to reconstruct phylogenies. (b) It alternates inter- and intra-sequence reasoning blocks. (c) Inter-sequence reasoning uses long bidirectional convolutions to extract shared motifs; intra-sequence reasoning applies sparsified attention to maintain per-sequence context.

**Table 1:** Evolutionary reasoning benchmark comparing Phyla to existing protein language models (PLMs). Tree reconstruction performance on TreeBase and TreeFam is evaluated using normalized Robinson-Foulds distance (normRF), where lower values indicate better agreement with ground truth phylogenies. Clustering performance at the class, order, family, genus, and species levels is measured using homogeneity scores, with higher values indicating more accurate unsupervised grouping according to known taxonomic labels. For other clustering metrics see Appendix A.6.

Model	TreeBase ↓	TreeFam ↓	Class ↑	Order ↑	Family ↑	Genus ↑	Species ↑
Hamming Distance	0.7466	0.7509	–	–	–	–	–
ESM2 (650M)	0.7809	0.6669	0.6378	0.6616	0.6838	0.7096	0.7542
ESM2 (3B)	0.7947	0.6729	0.5456	0.5624	0.5688	0.5873	0.6666
ESM3 (1.4B)	0.8139	0.7170	0.6067	0.6314	0.6596	0.6712	0.7212
ESM C (300M)	0.7680	0.7077	0.5743	0.6042	0.6223	0.6684	0.7145
ESM C (600M)	0.7990	0.7252	0.6106	0.6600	0.6577	0.7086	0.7522
Evo 2 (7B)	0.8372	0.8406	0.5002	0.5383	0.5521	0.5511	0.6398
ProGen2-Large (2.7B)	0.7747	0.6752	0.6049	0.6469	0.6641	0.7134	0.7505
ProGen2-XLarge (6.4B)	0.8624	0.8249	0.5157	0.5466	0.5705	0.6130	0.6800
***Phyla (24M)***	***0.7269***	***0.5777***	***0.7096***	***0.7646***	***0.8749***	***0.9267***	***0.9821***

**Table 2:** Ablation study on Phyla’s architecture and training objectives. Tree reconstruction is evaluated on TreeBase and TreeFam using normalized Robinson-Foulds distance (normRF), where lower values indicate better alignment with ground truth trees. Functional prediction is assessed on ProteinGym using Spearman correlation (higher is better). Taxonomic clustering is evaluated at five hierarchical levels—class, order, family, genus, and species—using homogeneity scores, with higher values indicating more taxonomically consistent clusters. Additional clustering metrics are reported in Appendix A.5.

Model	TreeBase ↓	TreeFam ↓	ProteinGym ↑	Class ↑	Order ↑	Family ↑	Genus ↑	Species ↑
Phyla-MLM	0.8291	0.7892	0.5536	0.6613	0.7554	0.8028	0.8712	0.9495
Phyla-NoAttention	0.8556	0.7839	0.4377	0.6929	0.7427	0.7830	0.8227	0.9192
**Phyla**	***0.7269***	***0.5777***	***0.6380***	***0.7096***	***0.7646***	***0.8749***	***0.9267***	***0.9821***

## Data Availability

Code to run Phyla can be found in the project github.
