# Supplementary material for: Evolutionary Reasoning Does Not Arise in Standard Usage of Protein Language Models
Source: bioRxiv. 2025 Oct 23:2025.01.17.633626. Originally published 2025 Jan 22. Preprint. [Version 3] doi: 10.1101/2025.01.17.633626 (PMC11785049; doi:10.1101/2025.01.17.633626)
Supplement: Supplement 1 [file NIHPP2025.01.17.633626v3-supplement-1.pdf]

bioRxiv preprint doi: https://doi.org/10.1101/2025.01.17.633626; this version posted October 24, 2025. The copyright holder for this preprint (which was not certified by peer review) is the author/funder, who has granted bioRxiv a license to display the preprint in perpetuity. It is made available under aCC-BY 4.0 International license.

A Technical Appendices and Supplementary Material

Table 3: Information about the 1,533 TreeBase evaluation datasets.

Dataset Metric	Min	Mean	Max	Standard Deviation
Average Sequence Length	5	322	3658	288
Number of Sequences	4	53	1284	65

Table 4: Information about the 9,586 TreeFam evaluation datasets

Dataset Metric	Min	Mean	Max	Standard Deviation
Average Sequence Length	40	599	31926	687
Number of Sequences	4	33	375	34

A.1 Tree reasoning and Protein Gym benchmark

A.1.1 Tree reasoning benchmark

Tree Reconstruction. We evaluate tree reconstruction performance using two external datasets: TreeBase Piel & Tannen (2009) and TreeFam Li et al. (2006). TreeBase is a curated repository of 12,817 phylogenetic trees from peer-reviewed studies, along with associated sequence data contributed by users. After filtering for trees with complete and parseable sequence information, we retained 1,533 trees for analysis. TreeFam is a database of gene family trees focused on animal genomes. We extracted 9,586 gene trees along with their corresponding protein sequences. To evaluate reconstruction quality, we computed the normalized Robinson-Foulds distance (lower is better) between the predicted and reference trees. We found that PHYLA had the lowest normalized Robinson-Foulds distance among all models. This difference was significant ($p < 0.05$, one-sided t-test, Table 5) for all models.

We also evaluate the overlap between the training corpus (OpenProteinSet) and the evaluation sets (TreeBase, TreeFam). We ran BLAST Altschul et al. (1990) between each tree in TreeBase and TreeFam and all the sequences in the training corpus using an e-value cutoff of 1e-5 and bitscore cutoff of 50. We found in TreeBase only 0.56% of evaluation trees had any match to the training set. The overall mean percent identity of sequences in TreeBase is 0.38%. In TreeFam only 3.38% of evaluation trees had any match to the training set. The overall mean percent identity of sequences in TreeFam is 0.23%. These results indicate minimal leakage and highlights the ability of PHYLA to generalize.

Table 5: P-values from paired t-tests comparing PHYLA with baseline PLMs on normalized Robinson-Foulds scores for TreeBase and TreeFam. Lower values indicate statistically significant improvements.

Baseline Model	TreeBase P-value	TreeFam P-value
ESM2 (650M)	6.78×10^{-16}	1.54×10^{-102}
ESMC (300M)	1.69×10^{-9}	7.95×10^{-222}
ESMC (600M)	4.61×10^{-27}	7.16×10^{-288}
ESM2 (3B)	7.72×10^{-24}	3.95×10^{-118}
ProGen2-Large (2.7B)	1.37×10^{-12}	4.63×10^{-112}
ProGen2-XLarge (6.4B)	7.99×10^{-99}	1.37×10^{-308}
ESM3 (1.4B)	3.20×10^{-40}	5.41×10^{-249}
Evo2 (7B)	4.71×10^{-65}	1.37×10^{-308}

Taxonomic Clustering Evaluation. We evaluate taxonomic structure using data from the Genome Taxonomy Database (GTDB), a high-quality and frequently updated resource for microbial taxonomy. GTDB taxonomic labels are derived by concatenating multiple sequence alignments of 120 marker

bioRxiv preprint doi: https://doi.org/10.1101/2025.01.17.633626; this version posted October 24, 2025. The copyright holder for this preprint (which was not certified by peer review) is the author/funder, who has granted bioRxiv a license to display the preprint in perpetuity. It is made available under aCC-BY 4.0 International license.

proteins per organism. We use Release 10-RS226 (released April 16, 2025), which contains 715,230 organisms.

To construct our evaluation dataset, we randomly sample 50 groups of 10 organisms each, ensuring that all organisms within a group share the same taxonomic label at either the class, order, family, genus, or species level depending on the benchmark. For each model, we compute organism-level embeddings as follows: (1) For baseline models, we embed each of the 120 marker proteins independently, then average the resulting embeddings to produce a single vector per organism. (2) For PHYLA, we batch all 500 organisms for each marker protein (e.g. protein 1) and compute embeddings jointly. We repeat this for all 120 proteins, then average across them to obtain organism-level embeddings. We then perform k-means clustering (as implemented in scikit-learn) with k=50, corresponding to the number of groups.

In the main text, we report results for one such sample of 500 organisms. However, we find that performance is consistent across clustering metrics (completeness, homogeneity, NMI) and across random seeds. We generate four additional random samples and report mean and standard deviation across these replicates. PHYLA achieves state-of-the-art performance in taxonomic clustering across all taxonomic levels. It is only outperformed by ProGen2-XLarge in completeness at the class level (Table 6).

A.1.2 Extended Benchmarking

Fine-tuned ESM2-650M. We fine-tuned the strongest baseline model, ESM2-650M, using two lightweight heads—a feed-forward network and a transformer head—trained with the same quartet loss as PHYLA, while freezing the backbone (as backpropagation through 650M parameters for trees with more than 100 leaves is computationally infeasible). Despite this targeted fine-tuning, the best variant achieved normalized Robinson–Foulds (normRF) scores of 0.77 on TreeBase and 0.79 on TreeFam, whereas PHYLA —trained end-to-end to reason jointly over sets of sequences—achieved 0.73 and 0.58, respectively. These results indicate that the performance gap cannot be attributed solely to evaluating PLMs in a zero-shot setting.

Input-Order Robustness. For each evaluation tree in TreeBase and TreeFam, PHYLA processes the full set of sequences in a single forward pass. Because the model is trained on randomly sampled and permuted sequence subsets, its predictions are invariant to input order. To verify this property, we randomly shuffled the sequence order for 250 trees from TreeBase five times each and measured the standard deviation in normalized RF scores. The mean deviation was 0.01, confirming that PHYLA's embeddings and resulting distance matrices are effectively deterministic with respect to sequence order.

Training signal comparison. To assess whether the choice of supervision signal influences performance, we compared two variants of PHYLA trained using (i) normalized Hamming distances computed from multiple sequence alignments (MSAs) and (ii) pairwise patristic distances extracted from trees reconstructed with FastTree on the same MSAs. Both variants achieved nearly identical performance on TreeBase and TreeFam benchmarks, with differences below 0.01 in normalized Robinson–Foulds distance (Table 7).

These results indicate that PHYLA does not simply replicate a specific distance metric but learns generalizable representations that capture consistent topological structure across distance definitions. In practice, Hamming distances serve as an efficient proxy for topological supervision without materially affecting model behavior or accuracy.

A.1.3 ProteinGym benchmark

The 83 ProteinGym datasets were chosen based on the memory constraint of a single 80GB H100 GPU. We stratified performance on the ProteinGym functional prediction benchmark across various levels of overlap between pre-training and evaluation datasets. We quantified overlap by running the Basic Local Alignment Search Tool (BLAST) algorithm between each representative sequence of the 83 ProteinGym datasets and each model's pre-training dataset (Altschul et al. (1990)). Overlap was calculated as the average percent similarity of output hits. We did not include the total number of hits when calculating the overlap metric in order to not further penalize models with larger pre-training datasets.

bioRxiv preprint doi: https://doi.org/10.1101/2025.01.17.633626; this version posted October 24, 2025. The copyright holder for this preprint (which was not certified by peer review) is the author/funder, who has granted bioRxiv a license to display the preprint in perpetuity. It is made available under aCC-BY 4.0 International license.

Table 6: Clustering evaluation metrics (mean ± std) across taxonomic levels. Standard deviation and mean across 5 random taxonomic group samples from GTDB.

**Taxonomic Level**	**Model**	Homogeneity	Completeness	NMI
Class	ESM2 (650M)	0.54 ± 0.05	0.64 ± 0.04	0.58 ± 0.05
	ESM3 (1.4B)	0.67 ± 0.01	0.71 ± 0.01	0.69 ± 0.01
	ESMC (300M)	0.55 ± 0.10	0.62 ± 0.10	0.58 ± 0.10
	ESMC (600M)	0.51 ± 0.02	0.59 ± 0.02	0.55 ± 0.02
	ProGen2-Large (2.7B)	0.67 ± 0.02	0.74 ± 0.02	0.70 ± 0.02
	ProGen2-XLarge (6.4B)	0.66 ± 0.04	**0.75 ± 0.03**	0.70 ± 0.03
	Evo2 (7B)	0.58 ± 0.02	0.67 ± 0.01	0.62 ± 0.01
	PHYLA (24M)	**0.69 ± 0.02**	0.73 ± 0.02	**0.71 ± 0.02**
Family	ESM2 (650M)	0.57 ± 0.05	0.68 ± 0.04	0.62 ± 0.04
	ESM3 (1.4B)	0.75 ± 0.02	0.78 ± 0.03	0.76 ± 0.02
	ESMC (300M)	0.59 ± 0.11	0.67 ± 0.10	0.63 ± 0.11
	ESMC (600M)	0.48 ± 0.04	0.61 ± 0.04	0.54 ± 0.04
	ProGen2-Large (2.7B)	0.74 ± 0.04	0.81 ± 0.03	0.77 ± 0.03
	ProGen2-XLarge (6.4B)	0.65 ± 0.07	0.76 ± 0.05	0.70 ± 0.06
	Evo2 (7B)	0.62 ± 0.05	0.72 ± 0.05	0.67 ± 0.05
	PHYLA (24M)	**0.85 ± 0.02**	**0.88 ± 0.02**	**0.86 ± 0.02**
Genus	ESM2 (650M)	0.64 ± 0.05	0.74 ± 0.04	0.68 ± 0.05
	ESM3 (1.4B)	0.83 ± 0.02	0.85 ± 0.02	0.84 ± 0.02
	ESMC (300M)	0.65 ± 0.10	0.73 ± 0.11	0.69 ± 0.10
	ESMC (600M)	0.51 ± 0.09	0.66 ± 0.06	0.58 ± 0.08
	ProGen2-Large (2.7B)	0.77 ± 0.06	0.85 ± 0.04	0.81 ± 0.05
	ProGen2-XLarge (6.4B)	0.68 ± 0.08	0.79 ± 0.05	0.73 ± 0.07
	Evo2 (7B)	0.74 ± 0.06	0.80 ± 0.04	0.77 ± 0.05
	PHYLA (24M)	**0.95 ± 0.02**	**0.97 ± 0.01**	**0.96 ± 0.01**
Order	ESM2 (650M)	0.57 ± 0.03	0.67 ± 0.03	0.62 ± 0.03
	ESM3 (1.4B)	0.72 ± 0.03	0.76 ± 0.02	0.74 ± 0.03
	ESMC (300M)	0.58 ± 0.10	0.65 ± 0.10	0.61 ± 0.10
	ESMC (600M)	0.51 ± 0.03	0.63 ± 0.02	0.57 ± 0.03
	ProGen2-Large (2.7B)	0.68 ± 0.04	0.76 ± 0.04	0.72 ± 0.04
	ProGen2-XLarge (6.4B)	0.65 ± 0.03	0.75 ± 0.03	0.69 ± 0.03
	Evo2 (7B)	0.60 ± 0.05	0.69 ± 0.03	0.64 ± 0.04
	PHYLA (24M)	**0.78 ± 0.03**	**0.81 ± 0.02**	**0.79 ± 0.02**
Species	ESM2 (650M)	0.69 ± 0.05	0.79 ± 0.04	0.73 ± 0.05
	ESM3 (1.4B)	0.85 ± 0.03	0.88 ± 0.03	0.87 ± 0.03
	ESMC (300M)	0.73 ± 0.06	0.82 ± 0.02	0.77 ± 0.04
	ESMC (600M)	0.52 ± 0.04	0.72 ± 0.04	0.60 ± 0.04
	ProGen2-Large (2.7B)	0.85 ± 0.04	0.90 ± 0.03	0.87 ± 0.03
	ProGen2-XLarge (6.4B)	0.73 ± 0.04	0.83 ± 0.03	0.77 ± 0.03
	Evo2 (7B)	0.82 ± 0.03	0.87 ± 0.03	0.84 ± 0.03
	PHYLA (24M)	**0.98 ± 0.01**	**0.99 ± 0.00**	**0.99 ± 0.01**

When comparing functional prediction performance between PHYLA and ESM2 on all 83 ProteinGym datasets, ESM2 significantly outperforms PHYLA by 0.14. However, on low-overlap regimes with less than 40% similarity to the model's pre-training dataset, PHYLA outperforms ESM2 by 0.03 as shown in Table 8 (Pearson (2013). Performance degrades on low-overlap regimes for all models, but PHYLA's performance degrades less. In addition, PHYLA occupies a lower overlap regime than ESM2, which is likely due to the smaller pre-training set used for PHYLA.

bioRxiv preprint doi: https://doi.org/10.1101/2025.01.17.633626; this version posted October 24, 2025. The copyright holder for this preprint (which was not certified by peer review) is the author/funder, who has granted bioRxiv a license to display the preprint in perpetuity. It is made available under aCC-BY 4.0 International license.

Table 7: Performance comparison when training with Hamming versus tree-based supervision signals.

**Training Signal**	**TreeBase (normRF)**	**TreeFam (normRF)**
Hamming Distance (original)	0.73	0.58
FastTree Distance	0.73	0.58

Table 8: **Functional Prediction Across Various Overlap Settings.** Model performance on predicting functional effects in ProteinGym stratified into low-overlap and high-overlap settings based on alignment overlap.

**Model**	**Low-Overlap**	**High-Overlap**	**All Datasets**
ESM2	0.59	**0.80**	**0.78**
PHYLA-MLM	0.53	0.61	0.55
PHYLA-NoAttention	0.40	0.53	0.44
PHYLA	**0.62**	0.68	0.64

A.2 Tree of Life analysis

To reconstruct the tree of life, we use a dataset of 3,083 organisms, each represented by the concatenated multiple sequence alignment (MSA) of 16 conserved ribosomal proteins Hug (2016). For input to PHYLA, we concatenate the raw amino acid sequences of all 16 ribosomal proteins per organism, yielding sequence lengths of approximately 6 million tokens. We process these sequences using PHYLA on a high-memory CPU machine (800 GB RAM), requiring approximately 16 hours of compute time for a full forward pass. We then compute pairwise distances between organism-level embeddings and apply the neighbor-joining algorithm to construct the resulting phylogenetic tree.

A.3 Tuberculosis analysis

Tuberculosis (TB) is one of the leading causes of death globally due to infectious disease World Health Organization (2024). A major challenge in controlling TB is its capacity to rapidly evolve drug resistance. Genomic surveillance has become a critical tool in addressing this issue: by tracking the evolutionary dynamics of TB, we can detect emerging resistance before it becomes widespread and adapt treatment strategies accordingly Thorpe et al. (2024). We use PHYLA to construct the first whole-genome phylogenetic tree of *Mycobacterium tuberculosis* isolates. Specifically, we analyze 151 complete TB genomes (around 4 Mb each) Marin et al. (2025). To generate organism-level representations, we divide each genome into non-overlapping 500 bp segments (shared across all 151 genomes), embed each chunk using PHYLA, and then average the resulting embeddings across the genome. This approach is well-suited to TB, which is a highly clonal organism with limited variation across isolates—making direct genome chunking and alignment across samples both feasible and meaningful Freschi et al. (2021). We compute pairwise distances between the organism-level embeddings and reconstruct the phylogenetic tree using the neighbor-joining algorithm (Figure 5).

PHYLA accurately reconstructs the major global lineages of *Mycobacterium tuberculosis*, recovering coherent clades corresponding to Lineages 1–6 and the recently described Lineage 8 Freschi et al. (2021). The topology is broadly consistent with established TB phylogenies, with early-branching Lineage 1 and distinct clusters for the derived East Asian (Lineage 2) and Euro-American (Lineage 4) groups. Deviations are observed in the relative positioning of Lineages 3 and 4, which appear slightly closer than expected. Given that PHYLA infers these relationships directly from sequence embeddings rather than explicit substitution models, the recovery of lineage-level structure without supervision underscores that the learned representation captures genuine evolutionary signal beyond surface sequence similarity.

bioRxiv preprint doi: https://doi.org/10.1101/2025.01.17.633626; this version posted October 24, 2025. The copyright holder for this preprint (which was not certified by peer review) is the author/funder, who has granted bioRxiv a license to display the preprint in perpetuity. It is made available under aCC-BY 4.0 International license.

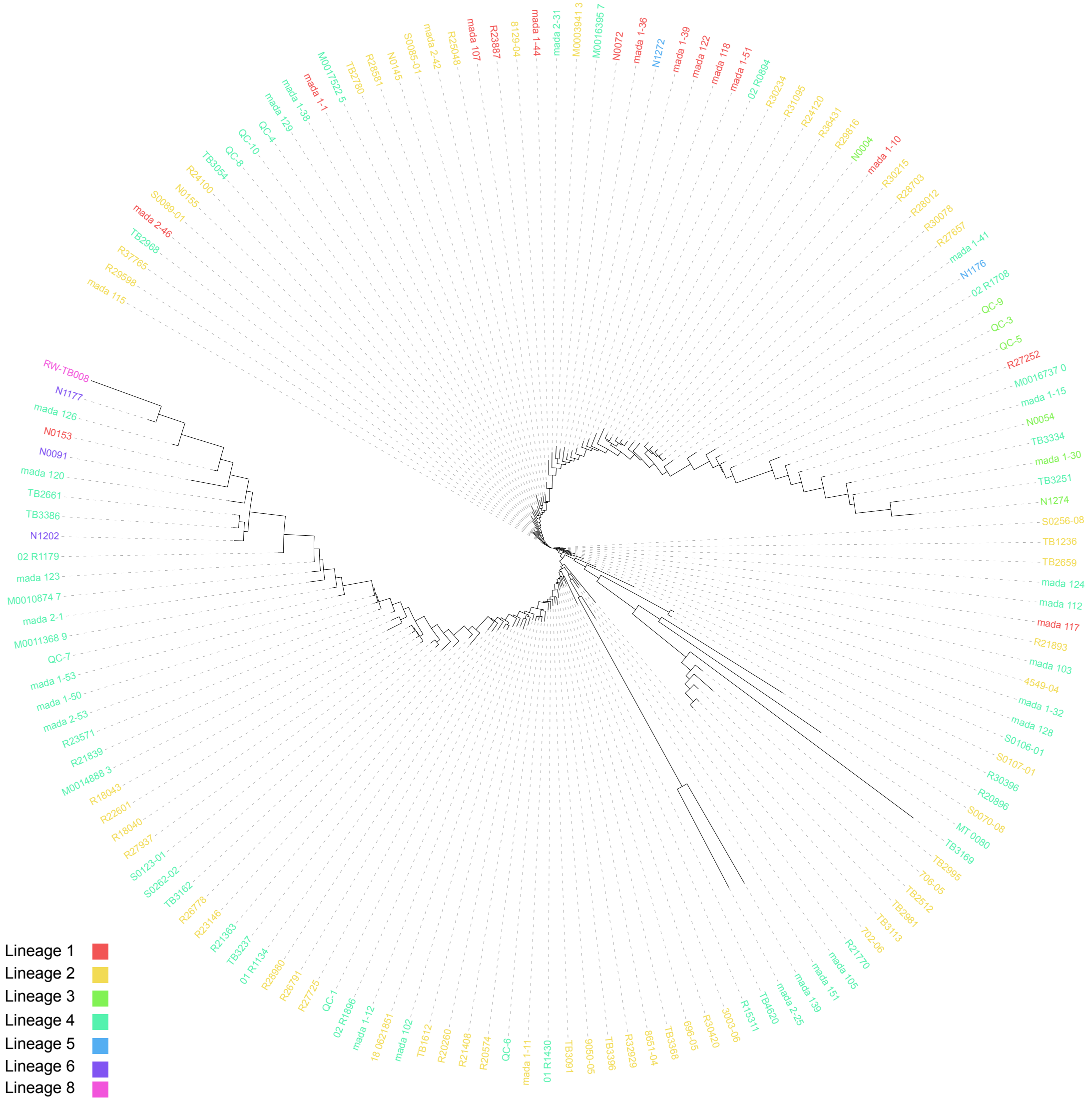

Figure 5: **PHYLA-generated tree of whole genome Tuberculosis (TB) isolates.** By comparing chunks of the genome of TB across 151 TB isolates, PHYLA constructed the first whole genome tree of TB. Labeled are the main lineages of TB (lineage2, lineage 3, and lineage 4).

A.4 Ablations

We retested our ablations, PHYLA-MLM trained with only the MLM loss and PHYLA-NoAttention trained without sparsified attention, on the extended taxonomic clustering dataset described in Section A.1.1. We found across all taxonomic levels, except class, PHYLA had the best performance across all metrics assessed. PHYLA also has significantly better tree reconstruction on TreeBase and TreeFam than all ablations (Table 10).

bioRxiv preprint doi: https://doi.org/10.1101/2025.01.17.633626; this version posted October 24, 2025. The copyright holder for this preprint (which was not certified by peer review) is the author/funder, who has granted bioRxiv a license to display the preprint in perpetuity. It is made available under aCC-BY 4.0 International license.

Table 9: Clustering evaluation metrics (mean ± std) for PHYLA variants across taxonomic levels. Standard deviation and mean across 5 random taxonomic group samples from GTDB.

**Taxonomic Level**	**Model**	Homogeneity	Completeness	NMI
Class	PHYLA-MLM	0.65 ± 0.02	0.69 ± 0.02	0.67 ± 0.02
	PHYLA-NoAttention	**0.69 ± 0.02**	**0.74 ± 0.01**	**0.71 ± 0.02**
	PHYLA	0.69 ± 0.02	0.73 ± 0.02	0.71 ± 0.02
Family	PHYLA-MLM	0.78 ± 0.02	0.80 ± 0.02	0.79 ± 0.02
	PHYLA-NoAttention	0.79 ± 0.02	0.83 ± 0.02	0.81 ± 0.02
	PHYLA	**0.85 ± 0.02**	**0.88 ± 0.02**	**0.87 ± 0.02**
Genus	PHYLA-MLM	0.89 ± 0.02	0.92 ± 0.02	0.90 ± 0.02
	PHYLA-NoAttention	0.85 ± 0.03	0.88 ± 0.02	0.87 ± 0.03
	PHYLA	**0.95 ± 0.02**	**0.97 ± 0.01**	**0.96 ± 0.01**
Order	PHYLA-MLM	0.74 ± 0.01	0.78 ± 0.01	0.76 ± 0.01
	PHYLA-NoAttention	0.74 ± 0.03	0.79 ± 0.02	0.76 ± 0.02
	PHYLA	**0.78 ± 0.03**	**0.81 ± 0.02**	**0.79 ± 0.02**
Species	PHYLA-MLM	0.94 ± 0.01	0.96 ± 0.01	0.95 ± 0.01
	PHYLA-NoAttention	0.92 ± 0.01	0.94 ± 0.01	0.93 ± 0.01
	PHYLA	**0.98 ± 0.01**	**0.99 ± 0.00**	**0.99 ± 0.01**

Table 10: P-values from paired t-tests comparing PHYLA with PHYLA ablations on normalized Robinson-Foulds scores for TreeBase and TreeFam. Lower p-values indicate stronger statistical significance.

**Baseline Model**	**TreeBase P-value**	**TreeFam P-value**
PHYLA-MLM	1.14×10^{-57}	0.0
PHYLA-NoAttention	1.94×10^{-91}	0.0

A.5 Evolution Reasoning Result

Please see Section A.1.1.

A.6 Recent Work

Evolutionary reasoning versus evolutionary modeling. Previous studies distinguish between fitting a generative model to observed sequence data and reasoning about the underlying fitness landscape that produced those sequences Weinstein et al. (2022); Ding et al. (2019). From these works, we know generative sequence models aim to match the marginal distribution of extant proteins (evolutionary modeling), whereas evolutionary reasoning seeks to recover the selective pressures and functional constraints driving those distributions.

AI for Phylogenetic Tree Construction. Recent years have seen a surge of AI-driven approaches for evolutionary-biology problems. For instance, PhyloVAE and ARTree Xie et al. (2025); Xie & Zhang (2023) generate plausible tree topologies from collections of existing phylogenies. GeoPhy and Phyloformer Mimori & Hamada (2023); Nesterenko et al. (2025) take a multiple sequence alignment (MSA) as input and infer both tree topology and branch lengths by maximizing the likelihood under a fixed substitution model. Other methods—such as DEPP and DeePhy Jiang et al. (2022); Mahapatra & Mukherjee (2025)—are designed to place unaligned query sequences into a reference phylogenetic tree or into existing triplets of sequences. In contrast, PHYLA focuses on a task that combines these capabilities: starting from unaligned sequences alone, it simultaneously infers a complete tree topology without requiring any prior alignment.

bioRxiv preprint doi: https://doi.org/10.1101/2025.01.17.633626; this version posted October 24, 2025. The copyright holder for this preprint (which was not certified by peer review) is the author/funder, who has granted bioRxiv a license to display the preprint in perpetuity. It is made available under aCC-BY 4.0 International license.

A.7 Evaluation on Masked Token Prediction

In addition to evaluating the ability of PHYLA and benchmark models to perform evolutionary reasoning, we also assess performance on a standard PLM task of masked-token prediction. PHYLA trained only with masked-language modeling attains 30% top-1 accuracy with reduced evolutionary reasoning performance; training with tree loss decreases accuracy to 11% but improves evolutionary reasoning performance (See PHYLA-MLM performance in 2 for evolutionary reasoning performance). Conventional PLMs (ESM-2 650M, ProGen2) achieve 45–55%. These results confirm a trade-off: per-token objectives favor single-sequence reconstruction, whereas the tree loss preserves cross-sequence signals essential for evolutionary reasoning.

A.8 Societal Impacts and Safety Concerns

Our method's ability to generalize beyond its training distribution can lead to more accurate identification of disease-causing variants in previously unseen protein families, potentially accelerating both basic research and the development of personalized therapeutics. However, because this is the first model that infers a phylogenetic tree directly from raw sequence data, there is a risk that users may overestimate its reliability and substitute it for established, alignment-based pipelines. Such overreliance could produce misleading evolutionary hypotheses or clinical interpretations if the model's assumptions and limitations are not carefully considered. To mitigate these dangers, we stress that our approach is intended as a complement to—rather than a replacement for—traditional phylogenetic methods, and that any high-stakes decisions should always include orthogonal validation.
