# Supplementary material for: Sequence Modeling Is Not Evolutionary Reasoning
Source: bioRxiv. 2025 Jun 27:2025.01.17.633626. Originally published 2025 Jan 22. Preprint. [Version 2] doi: 10.1101/2025.01.17.633626 (PMC11785049; doi:10.1101/2025.01.17.633626)
Supplement: Supplementary file 1 [file NIHPP2025.01.17.633626V2-supplement-1.pdf]

bioRxiv preprint doi: https://doi.org/10.1101/2025.01.17.633626; this version posted June 27, 2025. The copyright holder for this preprint (which was not certified by peer review) is the author/funder, who has granted bioRxiv a license to display the preprint in perpetuity. It is made available under aCC-BY 4.0 International license.

A Technical Appendices and Supplementary Material

Table 3: Table that shows information about the 1,533 TreeBase datasets used.

Dataset Metric	Min	Mean	Max	Standard Deviation
Average Sequence Length	5.0	322.4	3658.0	287.7
Number of Sequences	4.0	53.1	1284.0	65.1

Table 4: Table that shows information about the 9,586 TreeFam datasets used.

Dataset Metric	Min	Mean	Max	Standard Deviation
Average Sequence Length	40.0	599.1	31926.0	686.8
Number of Sequences	1.0	32.5	375.0	33.7

A.1 Phyla model architecture and training details

A.1.1 Model architecture details

The PHYLA architecture alternates between inter-sequence and intra-sequence reasoning blocks. Inter-sequence reasoning is performed using BiMamba layers, while intra-sequence reasoning is handled by sparsified attention layers Schiff et al. (2024). The current 24M-parameter PHYLA model comprises three inter-sequence blocks, each containing 16 BiMamba layers followed by a single sparsified attention layer. Each layer operates with a hidden dimension of 256.

A.1.2 Training details

PHYLA was trained on 3,321 high-quality multiple sequence alignments (MSAs) curated from the OpenProteinSet Ahdritz et al. (2023). Low-quality MSAs were removed using the filtering procedure described in EVE Frazer et al. (2021). For each MSA, we compute pairwise distances between sequences by counting the number of matching positions and dividing by the total alignment length. These normalized pairwise distances are used as supervision during training. We used MSA distances and not tree distances because the OpenProteinSet comprised of curated MSAs with no trees. To create trees we would have to run an algorithm on top of the MSA which could introduce biases while training.

The current model has 24M parameters and was trained on 1 80GB H100 GPU for 3 days with the AdamW optimizer using a 10,000 step linear warmup up to a learning rate of 1e-5 (Loshchilov & Hutter (2019)).

Adaptive Batch Size Sampling. We empirically determined that PHYLA can process input lengths up to 213,350 tokens on a 32 GB GPU and up to 302,350 tokens on a 48 GB GPU. For other GPU memory sizes, we used a linear model to estimate the maximum allowable input length. Given the length of the longest protein in the input, we computed the maximum number of sequences that could fit within the memory limit. To reduce overfitting, we randomly sampled the number of sequences between 5 and this maximum. Based on the sampled number of sequences, we then computed the largest batch size that would fit in memory. If a training step triggered an out-of-memory error, we automatically retried with both the number of sequences and the batch size reduced by half.

A.2 Tree reasoning and protein gym benchmark

A.2.1 Tree reasoning benchmark

Tree Reconstruction. We evaluate tree reconstruction performance using two external datasets: TreeBase Piel & Tannen (2009) and TreeFam Li et al. (2006). TreeBase is a curated repository of 12,817 phylogenetic trees from peer-reviewed studies, along with associated sequence data contributed by users. After filtering for trees with complete and parseable sequence information, we retained

bioRxiv preprint doi: https://doi.org/10.1101/2025.01.17.633626; this version posted June 27, 2025. The copyright holder for this preprint (which was not certified by peer review) is the author/funder, who has granted bioRxiv a license to display the preprint in perpetuity. It is made available under aCC-BY 4.0 International license.

1,533 trees for analysis. TreeFam is a database of gene family trees focused on animal genomes. We extracted 9,586 gene trees along with their corresponding protein sequences. For each method, we generated protein embeddings, computed pairwise distances between embeddings, and reconstructed trees using the neighbor-joining algorithm. To evaluate reconstruction quality, we computed the normalized Robinson-Foulds distance (lower is better) between the predicted and reference trees. We found that PHYLA had the lowest normalized Robinson-Foulds distance among all models. This difference was significant ($p < 0.05$, one-sided t-test, Table 5) for all models.

Table 5: P-values from paired t-tests comparing Phyla with baseline models on normalized Robinson-Foulds scores for TreeBase and TreeFam. Lower values indicate statistically significant improvements.

**Baseline Model**	**TreeBase P-value**	**TreeFam P-value**
ESM2 (650M)	6.78×10^{-16}	1.54×10^{-102}
ESMC (300M)	1.69×10^{-9}	7.95×10^{-222}
ESMC (600M)	4.61×10^{-27}	7.16×10^{-288}
ESM2 (3B)	7.72×10^{-24}	3.95×10^{-118}
ProGen2-Large (2.7B)	1.37×10^{-12}	4.63×10^{-112}
ProGen2-XLarge (6.4B)	7.99×10^{-99}	1.37×10^{-308}
ESM3 (1.4B)	3.20×10^{-40}	5.41×10^{-249}
Evo2 (7B)	4.71×10^{-65}	1.37×10^{-308}

Taxonomic Clustering Evaluation. We evaluate taxonomic structure using data from the Genome Taxonomy Database (GTDB), a high-quality and frequently updated resource for microbial taxonomy. GTDB taxonomic labels are derived by concatenating multiple sequence alignments of 120 marker proteins per organism. We use Release 10-RS226 (released April 16, 2025), which contains 715,230 organisms.

To construct our evaluation dataset, we randomly sample 50 groups of 10 organisms each, ensuring that all organisms within a group share the same taxonomic label at either the class, order, family, genus, or species level depending on the benchmark. For each model, we compute organism-level embeddings as follows: (1) For baseline models, we embed each of the 120 marker proteins independently, then average the resulting embeddings to produce a single vector per organism. (2) For PHYLA, we batch all 500 organisms for each marker protein (e.g., protein 1) and compute embeddings jointly. We repeat this for all 120 proteins, then average across them to obtain organism-level embeddings. We then perform k-means clustering (as implemented in scikit-learn) with k=50, corresponding to the number of groups.

In the main text, we report results for one such sample of 500 organisms. However, we find that performance is consistent across clustering metrics (completeness, homogeneity, NMI) and across random seeds. We generate four additional random samples and report mean and standard deviation across these replicates. Phyla achieves state-of-the-art performance in taxonomic clustering across all taxonomic levels. It is only outperformed by ProGen2-XLarge in completeness at the class level (Table 6).

bioRxiv preprint doi: https://doi.org/10.1101/2025.01.17.633626; this version posted June 27, 2025. The copyright holder for this preprint (which was not certified by peer review) is the author/funder, who has granted bioRxiv a license to display the preprint in perpetuity. It is made available under aCC-BY 4.0 International license.

Table 6: Clustering evaluation metrics (mean ± std) across taxonomic levels. Standard deviation and mean across 5 random taxonomic group samples from GTDB.

**Taxonomic Level**	**Model**	Homogeneity	Completeness	NMI
Class	ESM2 (650M)	0.536 ± 0.052	0.638 ± 0.038	0.582 ± 0.045
	ESM3 (1.4B)	0.674 ± 0.010	0.713 ± 0.014	0.693 ± 0.011
	ESMC (300M)	0.547 ± 0.097	0.622 ± 0.096	0.582 ± 0.097
	ESMC (600M)	0.505 ± 0.017	0.592 ± 0.018	0.545 ± 0.017
	ProGen2-Large (2.7B)	0.665 ± 0.023	0.743 ± 0.017	0.702 ± 0.020
	ProGen2-XLarge (6.4B)	0.655 ± 0.039	**0.746 ± 0.025**	0.697 ± 0.033
	Evo2 (7B)	0.583 ± 0.016	0.671 ± 0.009	0.624 ± 0.013
	Phyla	**0.689 ± 0.024**	0.725 ± 0.020	**0.706 ± 0.022**
Family	ESM2 (650M)	0.574 ± 0.045	0.683 ± 0.043	0.623 ± 0.044
	ESM3 (1.4B)	0.745 ± 0.024	0.784 ± 0.025	0.764 ± 0.024
	ESMC (300M)	0.591 ± 0.111	0.671 ± 0.097	0.628 ± 0.105
	ESMC (600M)	0.479 ± 0.041	0.614 ± 0.040	0.538 ± 0.041
	ProGen2-Large (2.7B)	0.735 ± 0.038	0.808 ± 0.030	0.770 ± 0.034
	ProGen2-XLarge (6.4B)	0.652 ± 0.068	0.758 ± 0.050	0.701 ± 0.060
	Evo2 (7B)	0.621 ± 0.051	0.716 ± 0.051	0.665 ± 0.051
	Phyla (24M)	**0.851 ± 0.020**	**0.878 ± 0.015**	**0.865 ± 0.017**
Genus	ESM2 (650M)	0.638 ± 0.053	0.736 ± 0.039	0.683 ± 0.047
	ESM3 (1.4B)	0.825 ± 0.017	0.851 ± 0.020	0.837 ± 0.018
	ESMC (300M)	0.654 ± 0.099	0.727 ± 0.109	0.688 ± 0.104
	ESMC (600M)	0.511 ± 0.088	0.664 ± 0.061	0.576 ± 0.080
	ProGen2-Large (2.7B)	0.774 ± 0.058	0.849 ± 0.041	0.810 ± 0.050
	ProGen2-XLarge (6.4B)	0.675 ± 0.078	0.788 ± 0.051	0.726 ± 0.067
	Evo2 (7B)	0.735 ± 0.059	0.801 ± 0.040	0.767 ± 0.050
	Phyla (24M)	**0.953 ± 0.015**	**0.968 ± 0.011**	**0.960 ± 0.013**
Order	ESM2 (650M)	0.572 ± 0.034	0.674 ± 0.030	0.619 ± 0.032
	ESM3 (1.4B)	0.724 ± 0.029	0.760 ± 0.024	0.741 ± 0.026
	ESMC (300M)	0.580 ± 0.095	0.652 ± 0.103	0.614 ± 0.098
	ESMC (600M)	0.514 ± 0.031	0.631 ± 0.021	0.567 ± 0.027
	ProGen2-Large (2.7B)	0.677 ± 0.043	0.760 ± 0.035	0.716 ± 0.040
	ProGen2-XLarge (6.4B)	0.648 ± 0.026	0.745 ± 0.030	0.693 ± 0.027
	Evo2 (7B)	0.598 ± 0.050	0.694 ± 0.031	0.643 ± 0.042
	Phyla (24M)	**0.779 ± 0.027**	**0.810 ± 0.021**	**0.794 ± 0.024**
Species	ESM2 (650M)	0.685 ± 0.050	0.789 ± 0.037	0.733 ± 0.045
	ESM3 (1.4B)	0.851 ± 0.031	0.882 ± 0.026	0.866 ± 0.029
	ESMC (300M)	0.730 ± 0.055	0.819 ± 0.021	0.771 ± 0.039
	ESMC (600M)	0.523 ± 0.036	0.717 ± 0.039	0.604 ± 0.037
	ProGen2-Large (2.7B)	0.845 ± 0.035	0.899 ± 0.025	0.871 ± 0.029
	ProGen2-XLarge (6.4B)	0.726 ± 0.038	0.830 ± 0.031	0.774 ± 0.034
	Evo2 (7B)	0.816 ± 0.028	0.874 ± 0.025	0.844 ± 0.027
	Phyla (24M)	**0.983 ± 0.007**	**0.992 ± 0.004**	**0.987 ± 0.005**

A.2.2 ProteinGym benchmark

The 83 ProteinGym datasets were chosen based on the memory constraint of a single 80GB H100 GPU. We stratified performance on the ProteinGym functional prediction benchmark across various levels of overlap between pre-training and evaluation datasets. We quantified overlap by running the Basic Local Alignment Search Tool (BLAST) algorithm between each representative sequence of the 83 ProteinGym datasets and each model's pre-training dataset (Altschul et al. (1990)). Overlap was calculated as the average percent similarity of output hits. We did not include the total number of hits

bioRxiv preprint doi: https://doi.org/10.1101/2025.01.17.633626; this version posted June 27, 2025. The copyright holder for this preprint (which was not certified by peer review) is the author/funder, who has granted bioRxiv a license to display the preprint in perpetuity. It is made available under aCC-BY 4.0 International license.

Table 7: **Functional Prediction Across Various Overlap Settings.** Model performance on predicting functional effects in ProteinGym stratified into low-overlap and high-overlap settings based on alignment overlap.

Model	Low-Overlap	High-Overlap	All Datasets
ESM2	0.5863	**0.8013**	**0.7754**
PHYLA-MLM	0.5277	0.6135	0.5535
PHYLA-NoAttention	0.3981	0.5297	0.4377
PHYLA	***0.6208***	*0.6778*	*0.6380*

when calculating the overlap metric in order to not further penalize models with larger pre-training datasets.

When comparing functional prediction performance between PHYLA and ESM2 on all 83 ProteinGym datasets, ESM2 significantly outperforms PHYLA by 0.137. However, on low-overlap regimes with less than 40% similarity to the model's pre-training dataset, PHYLA outperforms ESM2 by 0.034 as shown in Table 7 (Pearson (2013). Performance degrades on low-overlap regimes for all models, but PHYLA's performance degrades less. In addition, PHYLA occupies a lower overlap regime than ESM2, which is likely due to the smaller pre-training set used for PHYLA.

A.3 Tree of Life analysis

To reconstruct the tree of life, we use a dataset of 3,083 organisms, each represented by the concatenated multiple sequence alignment (MSA) of 16 conserved ribosomal proteins Hug (2016). For input to PHYLA, we concatenate the raw amino acid sequences of all 16 ribosomal proteins per organism, yielding sequence lengths of approximately 6 million tokens. We process these sequences using PHYLA on a high-memory CPU machine (800 GB RAM), requiring approximately 16 hours of compute time for a full forward pass. We then compute pairwise distances between organism-level embeddings and apply the neighbor-joining algorithm to construct the resulting phylogenetic tree.

A.4 Tuberculosis analysis

Tuberculosis (TB) is one of the leading causes of death globally due to infectious disease World Health Organization (2024). A major challenge in controlling TB is its capacity to rapidly evolve drug resistance. Genomic surveillance has become a critical tool in addressing this issue: by tracking the evolutionary dynamics of TB, we can detect emerging resistance before it becomes widespread and adapt treatment strategies accordingly Thorpe et al. (2024). We use Phyla to construct the first whole-genome phylogenetic tree of Mycobacterium tuberculosis isolates. Specifically, we analyze 92 complete TB genomes (4 Mb each) Marin et al. (2025). To generate organism-level representations, we divide each genome into non-overlapping 500 bp segments (shared across all 92 genomes), embed each chunk using Phyla, and then average the resulting embeddings across the genome. This approach is well-suited to TB, which is a highly clonal organism with limited variation across isolates—making direct genome chunking and alignment across samples both feasible and meaningful Freschi et al. (2021). We compute pairwise distances between the organism-level embeddings and reconstruct the phylogenetic tree using the neighbor-joining algorithm (Figure 5).

PHYLA successfully clusters Mycobacterium tuberculosis isolates according to their known lineages, including correctly grouping isolates from Lineage 4 and Lineage 2 Freschi et al. (2021). The tree also reflects known ambiguity between Lineages 2 and 3, a pattern previously reported in the TB phylogenetics literature Freschi et al. (2021). However, PHYLA places Lineage 4 closer to Lineage 2 than expected. In established phylogenies, Lineage 4 is believed to have diverged earlier, suggesting a discrepancy from the accepted evolutionary history of TB. This deviation may result from artifacts introduced during genome chunking, or it may point to a previously unrecognized evolutionary signal. Investigating the cause of this discrepancy—and whether it reflects a biological insight or a modeling artifact—is an important direction for future work.

bioRxiv preprint doi: https://doi.org/10.1101/2025.01.17.633626; this version posted June 27, 2025. The copyright holder for this preprint (which was not certified by peer review) is the author/funder, who has granted bioRxiv a license to display the preprint in perpetuity. It is made available under aCC-BY 4.0 International license.

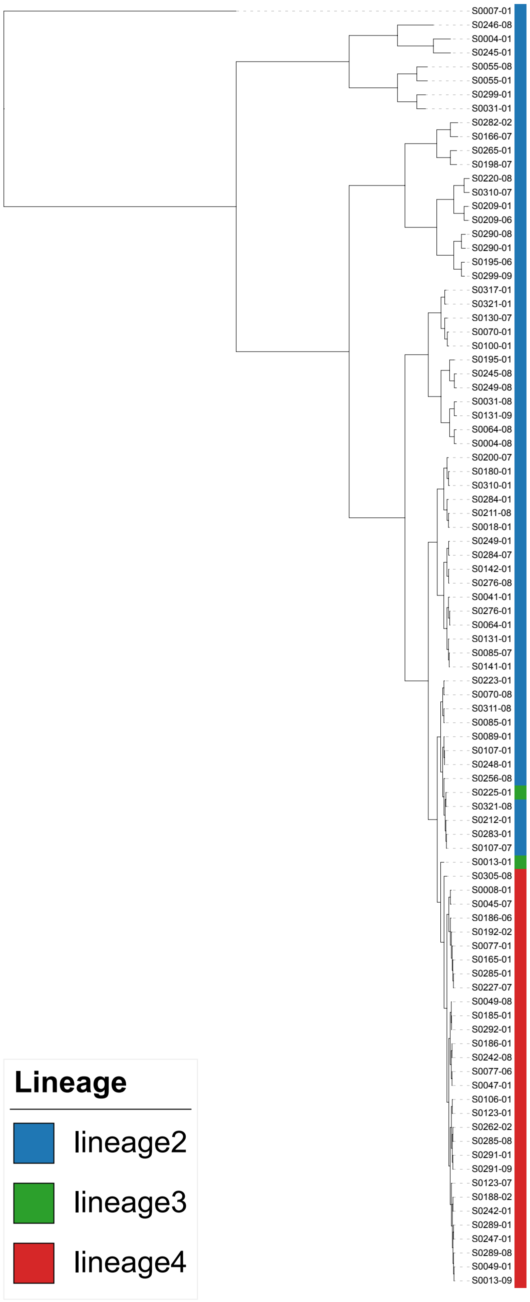

Figure 5: **Phyla generated tree of whole genome Tuberuclosis (TB) isolates.** By comparing chunks of the genome of TB across 92 TB isolates, Phyla constructed the first-whole genome tree of TB. Labeled are the main lineages of TB (lineage2, lineage 3, and lineage 4).

A.5 Ablations

We retested our ablations, Phyla-MLM trained with only the MLM loss and Phyla-NoAttention trained without sparsified attention, on the extended taxonomic clustering dataset described in Section A.2.1. We found across all taxonomic levels, except class, PHYLA had the best performance across all metrics assessed. PHYLA also has significantly better tree reconstruction in TreeBase and TreeFam than all ablations (Table 9).

bioRxiv preprint doi: https://doi.org/10.1101/2025.01.17.633626; this version posted June 27, 2025. The copyright holder for this preprint (which was not certified by peer review) is the author/funder, who has granted bioRxiv a license to display the preprint in perpetuity. It is made available under aCC-BY 4.0 International license.

Table 8: Clustering evaluation metrics (mean ± std) for Phyla variants across taxonomic levels. Standard deviation and mean across 5 random taxonomic group samples from GTDB.

**Taxonomic Level**	**Model**	Homogeneity	Completeness	NMI
Class	Phyla-MLM	0.651 ± 0.018	0.686 ± 0.021	0.668 ± 0.019
	Phyla-NoAttention	**0.690 ± 0.018**	**0.740 ± 0.011**	**0.714 ± 0.015**
	Phyla	0.689 ± 0.024	0.725 ± 0.020	0.706 ± 0.022
Family	Phyla-MLM	0.775 ± 0.022	0.804 ± 0.017	0.789 ± 0.019
	Phyla-NoAttention	0.790 ± 0.022	0.831 ± 0.019	0.810 ± 0.021
	Phyla	**0.851 ± 0.020**	**0.878 ± 0.015**	**0.865 ± 0.017**
Genus	Phyla-MLM	0.891 ± 0.016	0.915 ± 0.015	0.903 ± 0.016
	Phyla-NoAttention	0.849 ± 0.029	0.881 ± 0.024	0.865 ± 0.026
	Phyla	**0.953 ± 0.015**	**0.968 ± 0.011**	**0.960 ± 0.013**
Order	Phyla-MLM	0.739 ± 0.014	0.778 ± 0.012	0.758 ± 0.012
	Phyla-NoAttention	0.736 ± 0.029	0.787 ± 0.016	0.761 ± 0.023
	Phyla	**0.779 ± 0.027**	**0.810 ± 0.021**	**0.794 ± 0.024**
Species	Phyla-MLM	0.944 ± 0.011	0.963 ± 0.013	0.954 ± 0.012
	Phyla-NoAttention	0.924 ± 0.011	0.943 ± 0.011	0.934 ± 0.010
	Phyla	**0.983 ± 0.007**	**0.992 ± 0.004**	**0.987 ± 0.005**

Table 9: P-values from paired t-tests comparing Phyla with Phyla ablations on normalized Robinson-Foulds scores for TreeBase and TreeFam. Lower p-values indicate stronger statistical significance.

**Baseline Model**	**TreeBase P-value**	**TreeFam P-value**
PHYLA-MLM	1.14×10^{-57}	0.0
PHYLA-NoAttention	1.94×10^{-91}	0.0

A.6 Evolution Reasoning Result

Please see Section A.2.1.

A.7 Recent Work

Evolutionary reasoning versus evolutionary modeling. Previous studies distinguish between fitting a generative model to observed sequence data and reasoning about the underlying fitness landscape that produced those sequences Weinstein et al. (2022); Ding et al. (2019). From these works, we know generative sequence models aim to match the marginal distribution of extant proteins (evolutionary modeling), whereas evolutionary reasoning seeks to recover the selective pressures and functional constraints driving those distributions.

AI for Phylogenetic Tree Construction. Recent years have seen a surge of AI-driven approaches for evolutionary-biology problems. For instance, PhyloVAE and ARTree Xie et al. (2025); Xie & Zhang (2023) generate plausible tree topologies from collections of existing phylogenies. GeoPhy and Phyloformer Mimori & Hamada (2023); Nesterenko et al. (2025) take an input multiple-sequence alignment (MSA) and infer both tree topology and branch lengths by maximizing the likelihood under a fixed substitution model. Other methods—such as DEPP and DeePhy Jiang et al. (2022); Mahapatra & Mukherjee (2025)—are designed to place unaligned query sequences into a reference phylogenetic tree or into existing triplets of sequences. In contrast, PHYLA focuses on a task that combines these capabilities: starting from unaligned sequences alone, it simultaneously infers a complete tree topology without requiring any prior alignment.

bioRxiv preprint doi: https://doi.org/10.1101/2025.01.17.633626; this version posted June 27, 2025. The copyright holder for this preprint (which was not certified by peer review) is the author/funder, who has granted bioRxiv a license to display the preprint in perpetuity. It is made available under aCC-BY 4.0 International license.

A.8 Limitations and Future Directions

A primary limitation of our approach is its reliance on an external distance-based algorithm (e.g., neighbor-joining) for tree reconstruction. While this may introduce biases, we apply the same reconstruction procedure across all models to ensure fair comparison. Additionally, because PHYLA operates in Euclidean space, it may be suboptimal for modeling hierarchical structures that are more naturally represented in non-Euclidean (e.g., hyperbolic) spaces. Incorporating hyperbolic embeddings into future versions of PHYLA may further enhance its performance. Finally, while we benchmarked against simple baselines such as Hamming distance, we did not compare PHYLA to the full suite of classical phylogenetic pipelines. This choice was deliberate: PHYLA is not intended as a drop-in replacement for existing phylogenetic methods, but rather as a first step toward a new paradigm for evolutionary reasoning with protein language models. Beyond hyperbolic embeddings, future work includes moving away from tree reconstruction with the neighbor-joining algorithm and having PHYLA construct its own tree topologies based on the protein embeddings it generates.

Societal Impacts and Safety Concerns. Our method's ability to generalize beyond its training distribution can lead to more accurate identification of disease-causing variants in previously unseen protein families, potentially accelerating both basic research and the development of personalized therapeutics. However, because this is the first model that infers a phylogenetic tree directly from raw sequence data, there is a risk that users may overestimate its reliability and substitute it for established, alignment-based pipelines. Such overreliance could produce misleading evolutionary hypotheses or clinical interpretations if the model's assumptions and limitations are not carefully considered. To mitigate these dangers, we stress that our approach is intended as a complement to—rather than a replacement for—traditional phylogenetic methods, and that any high-stakes decisions (e.g., in clinical genomics) should always include orthogonal validation.
