# Supplementary material for: Phyla: Towards a foundation model for phylogenetic inference
Source: bioRxiv. 2025 Jan 22:2025.01.17.633626. Preprint. [Version 1] doi: 10.1101/2025.01.17.633626 (PMC11785049; doi:10.1101/2025.01.17.633626)
Supplement: Supplementary file 1 [file NIHPP2025.01.17.633626V1-supplement-1.pdf]

bioRxiv preprint doi: https://doi.org/10.1101/2025.01.17.633626; this version posted January 22, 2025. The copyright holder for this preprint (which was not certified by peer review) is the author/funder, who has granted bioRxiv a license to display the preprint in perpetuity. It is made available under aCC-BY 4.0 International license.

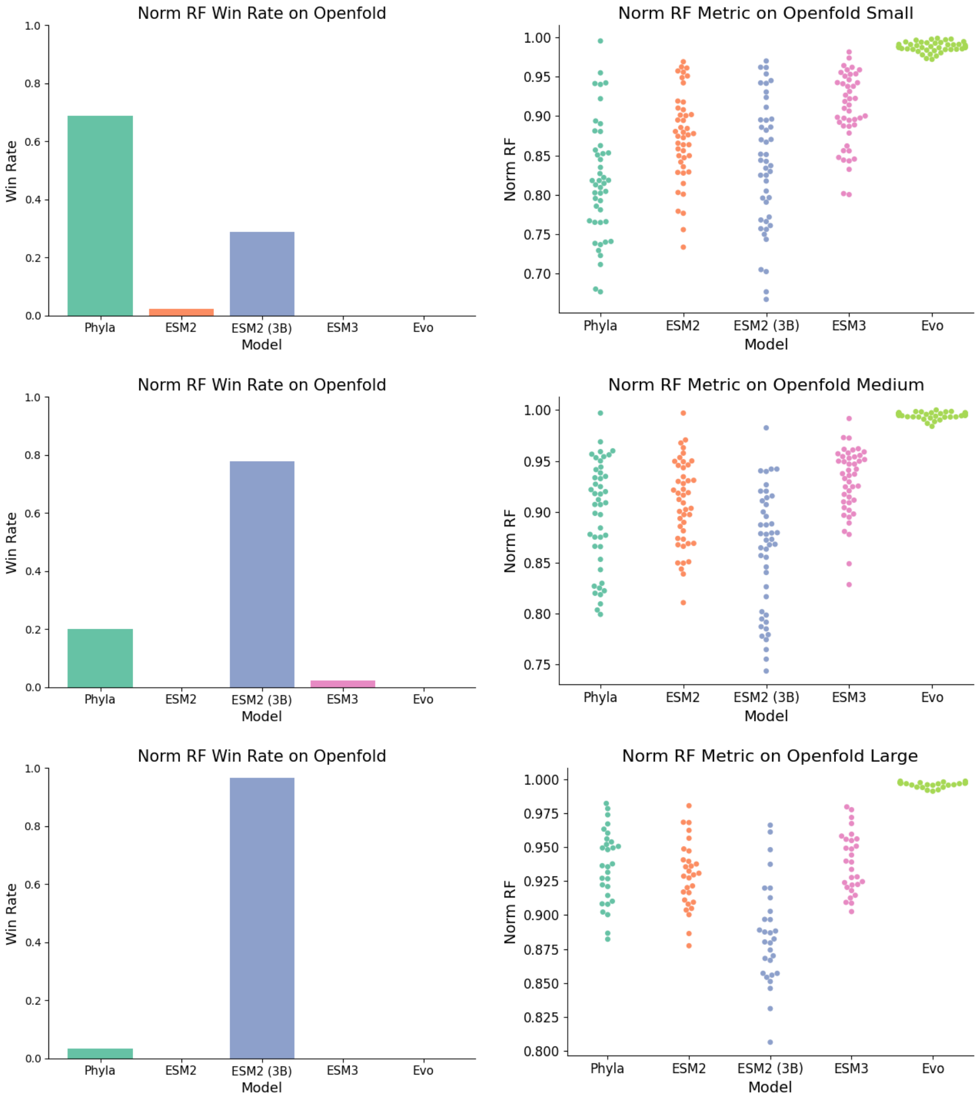

Figure S1: (a) Win rate (higher value is better) of normRF metric values for the Openfold Small (0 to 1000 sequences) evaluation set for Phyla vs ESM models vs Evo. (b) Swarm plot of normRF metric (lower is better) values for all datasets in the Openfold Small (0 to 1000 sequences) evaluation set for Phyla vs ESM models vs Evo. (c) Win rate (higher value is better) of normRF metric values for the Openfold Medium (1000 to 2000 sequences) evaluation set for Phyla vs ESM models vs Evo. (d) Swarm plot of normRF metric (lower is better) values for all datasets in the Openfold Medium (1000 to 2000 sequences) evaluation set for Phyla vs ESM models vs Evo. (e) Win rate (higher value is better) of normRF metric values for the Openfold Large (2000+ sequences) evaluation set for Phyla vs ESM models vs Evo. (f) Swarm plot of normRF metric (lower is better) values for all datasets in the Openfold Large (2000+ sequences) evaluation set for Phyla vs ESM models vs Evo.

bioRxiv preprint doi: https://doi.org/10.1101/2025.01.17.633626; this version posted January 22, 2025. The copyright holder for this preprint (which was not certified by peer review) is the author/funder, who has granted bioRxiv a license to display the preprint in perpetuity. It is made available under aCC-BY 4.0 International license.

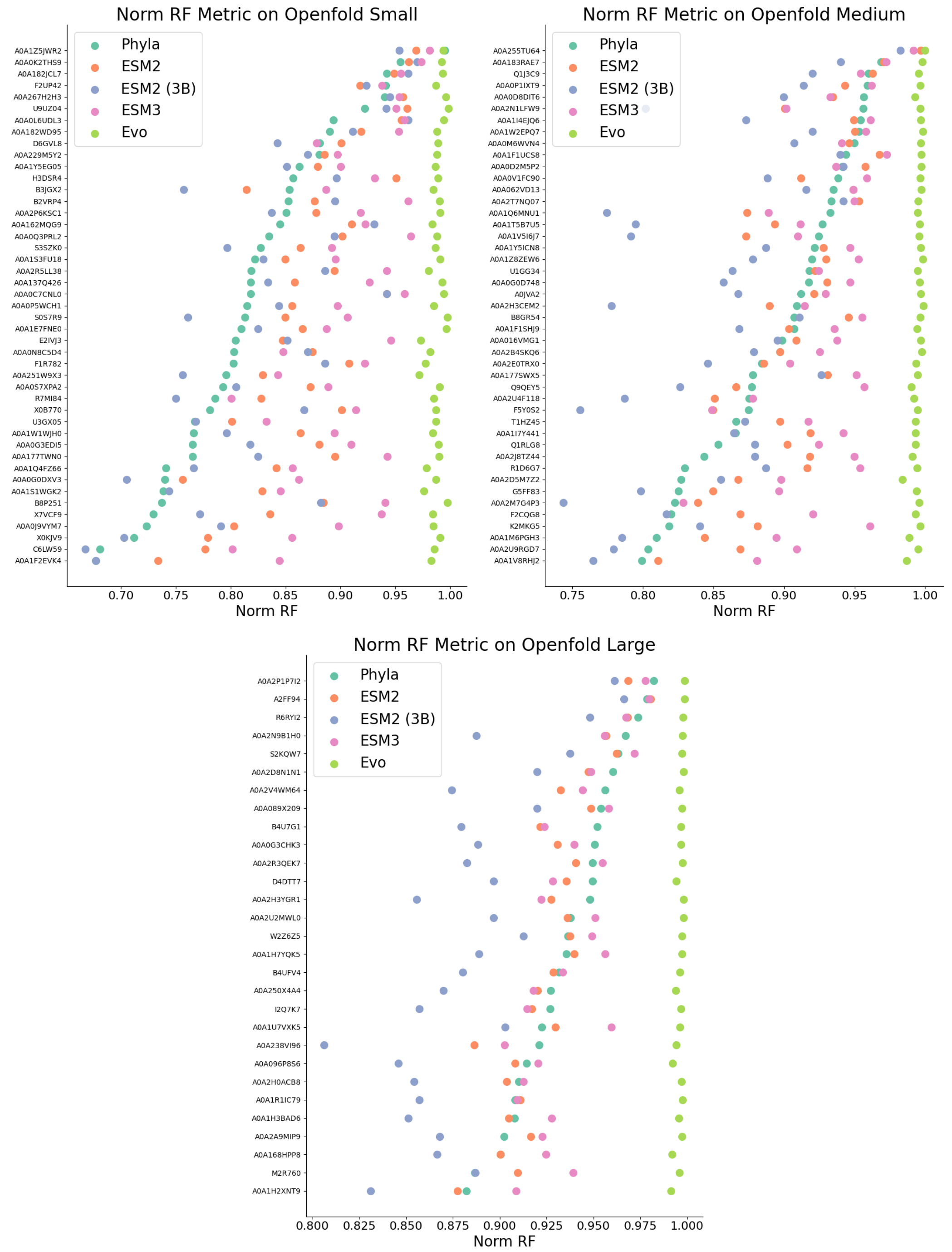

Figure S2: (a) Norm RF metric (lower, i.e. to the left, is better) values for all datasets in the Openfold Small (0 to 1000 sequences) evaluation set for Phyla vs ESM models vs Evo. (b) Norm RF metric (lower, i.e. to the left, is better) values for all datasets in the Openfold Medium (1000 to 2000 sequences) evaluation set for Phyla vs ESM models vs Evo. (c) Norm RF metric (lower, i.e. to the left, is better) values for all datasets in the Openfold Large (2000+ sequences) evaluation set for Phyla vs ESM models vs Evo.

bioRxiv preprint doi: https://doi.org/10.1101/2025.01.17.633626; this version posted January 22, 2025. The copyright holder for this preprint (which was not certified by peer review) is the author/funder, who has granted bioRxiv a license to display the preprint in perpetuity. It is made available under aCC-BY 4.0 International license.

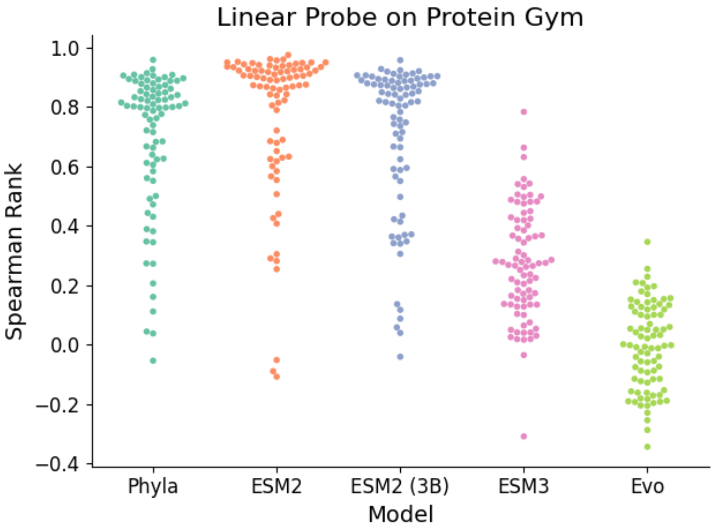

Figure S3: (a) Swarm plot of Spearman correlation (higher is better) values using linear probe calculation method for 83 datasets in the Protein Gym evaluation set for Phyla vs ESM models vs Evo.

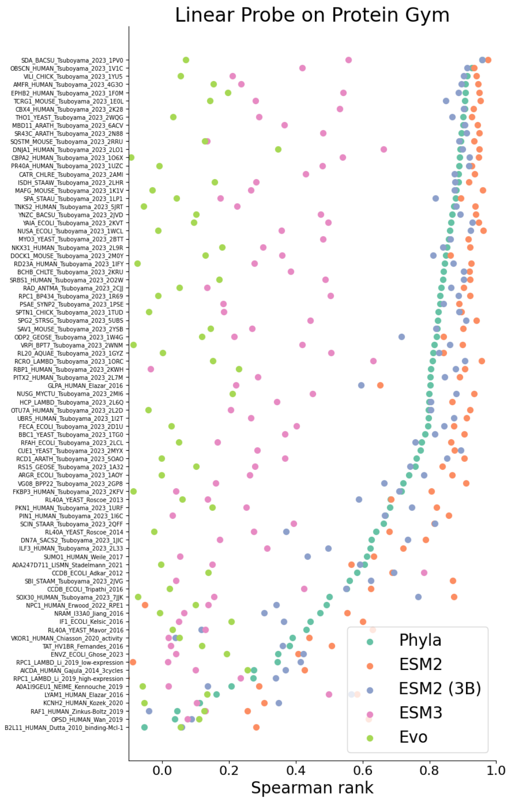

Figure S4: (a) Spearman correlation metric (higher, i.e. to the right, is better) values using linear probe calculation method for 83 datasets in the Protein Gym evaluation set for Phyla vs ESM models vs Evo.

bioRxiv preprint doi: https://doi.org/10.1101/2025.01.17.633626; this version posted January 22, 2025. The copyright holder for this preprint (which was not certified by peer review) is the author/funder, who has granted bioRxiv a license to display the preprint in perpetuity. It is made available under aCC-BY 4.0 International license.

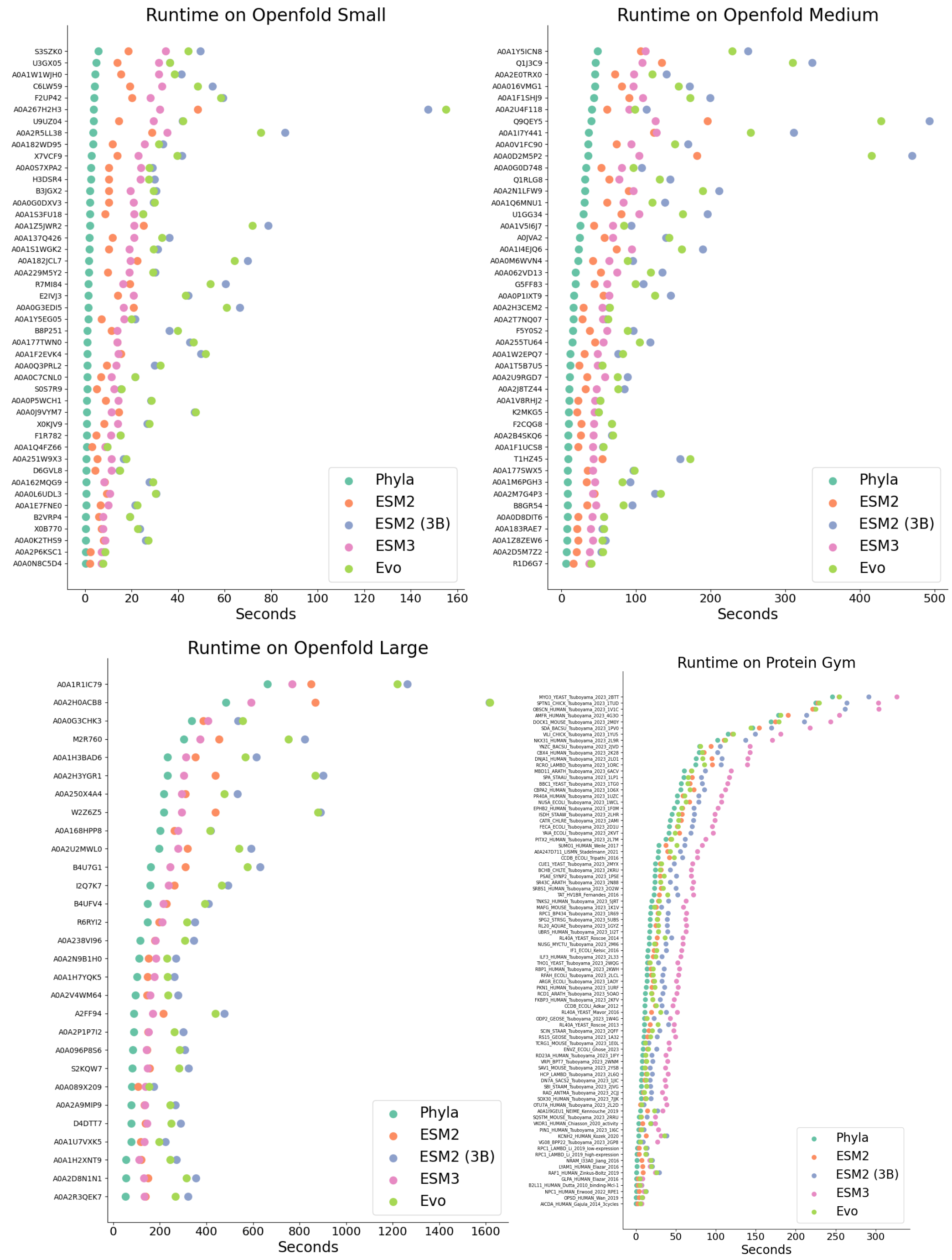

Figure S5: (a) Runtime metric (lower, i.e. to the left, is better) in seconds for all datasets in the Openfold Small (0 to 1000 sequences) evaluation set for Phyla vs ESM models vs Evo. (b) Runtime metric (lower, i.e. to the left, is better) in seconds for all datasets in the Openfold Medium (1000 to 2000 sequences) evaluation set for Phyla vs ESM models vs Evo. (c) Runtime metric (lower, i.e. to the left, is better) in seconds for all datasets in the Openfold Large (2000+ sequences) evaluation set for Phyla vs traditional method (in green) vs ESM models. (d) Runtime metric (lower, i.e. to the left, is better) in seconds for 83 datasets in the Protein Gym evaluation set for Phyla vs ESM models vs Evo.

bioRxiv preprint doi: https://doi.org/10.1101/2025.01.17.633626; this version posted January 22, 2025. The copyright holder for this preprint (which was not certified by peer review) is the author/funder, who has granted bioRxiv a license to display the preprint in perpetuity. It is made available under aCC-BY 4.0 International license.

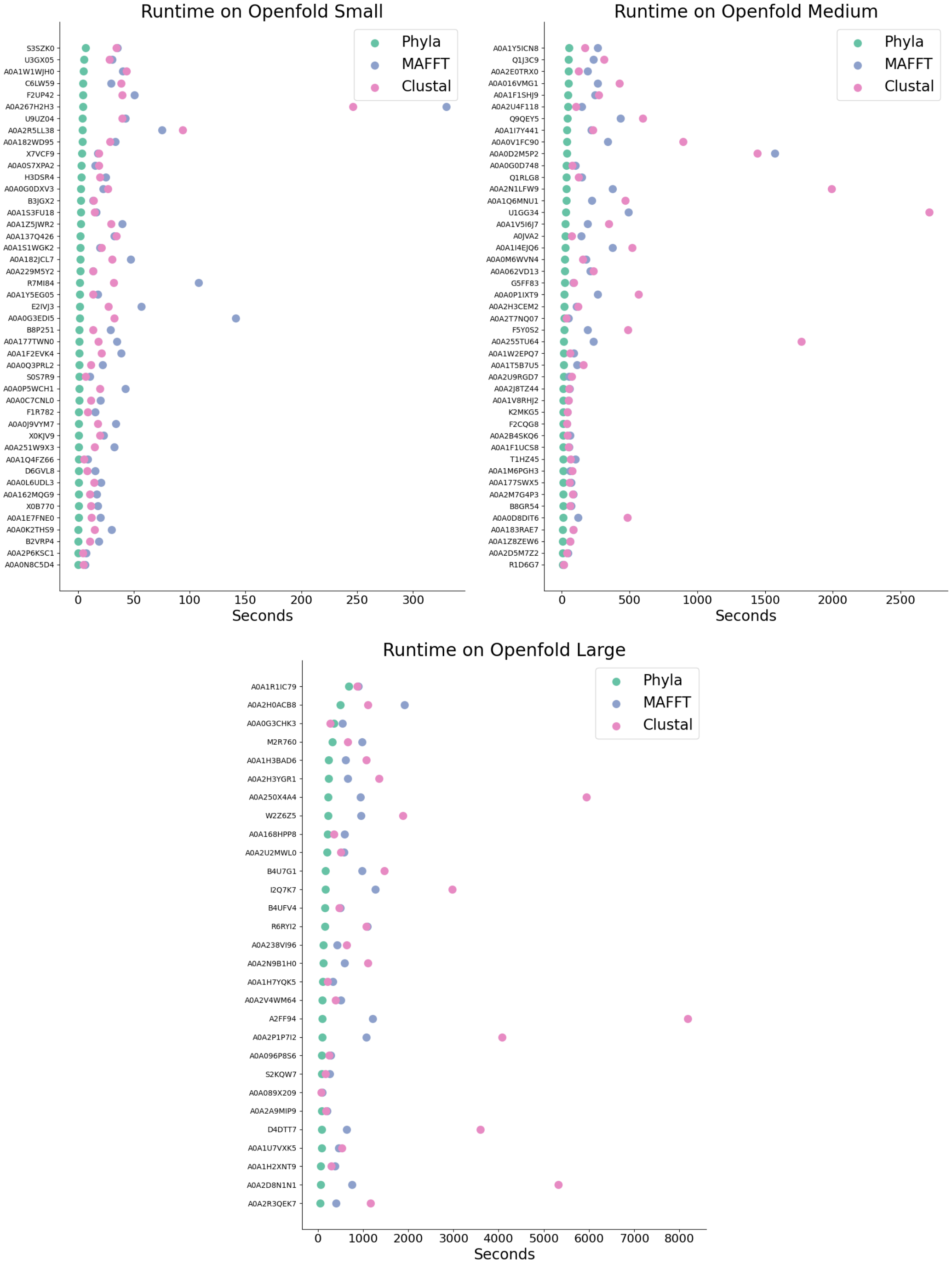

Figure S6: (a) Runtime metric (lower, i.e. to the left, is better) in seconds for all datasets in the Openfold Small (0 to 1000 sequences) evaluation set for Phyla vs traditional methods. (b) Runtime metric (lower, i.e. to the left, is better) in seconds for all datasets in the Openfold Medium (1000 to 2000 sequences) evaluation set for Phyla vs traditional methods. (c) Runtime metric (lower, i.e. to the left, is better) in seconds for all datasets in the Openfold Large (2000+ sequences) evaluation set for for Phyla vs traditional methods. *Note*: “traditional method” consists of taking sequences, aligning those sequences to create an MSA, and then using that MSA to construct a tree.

bioRxiv preprint doi: https://doi.org/10.1101/2025.01.17.633626; this version posted January 22, 2025. The copyright holder for this preprint (which was not certified by peer review) is the author/funder, who has granted bioRxiv a license to display the preprint in perpetuity. It is made available under aCC-BY 4.0 International license.

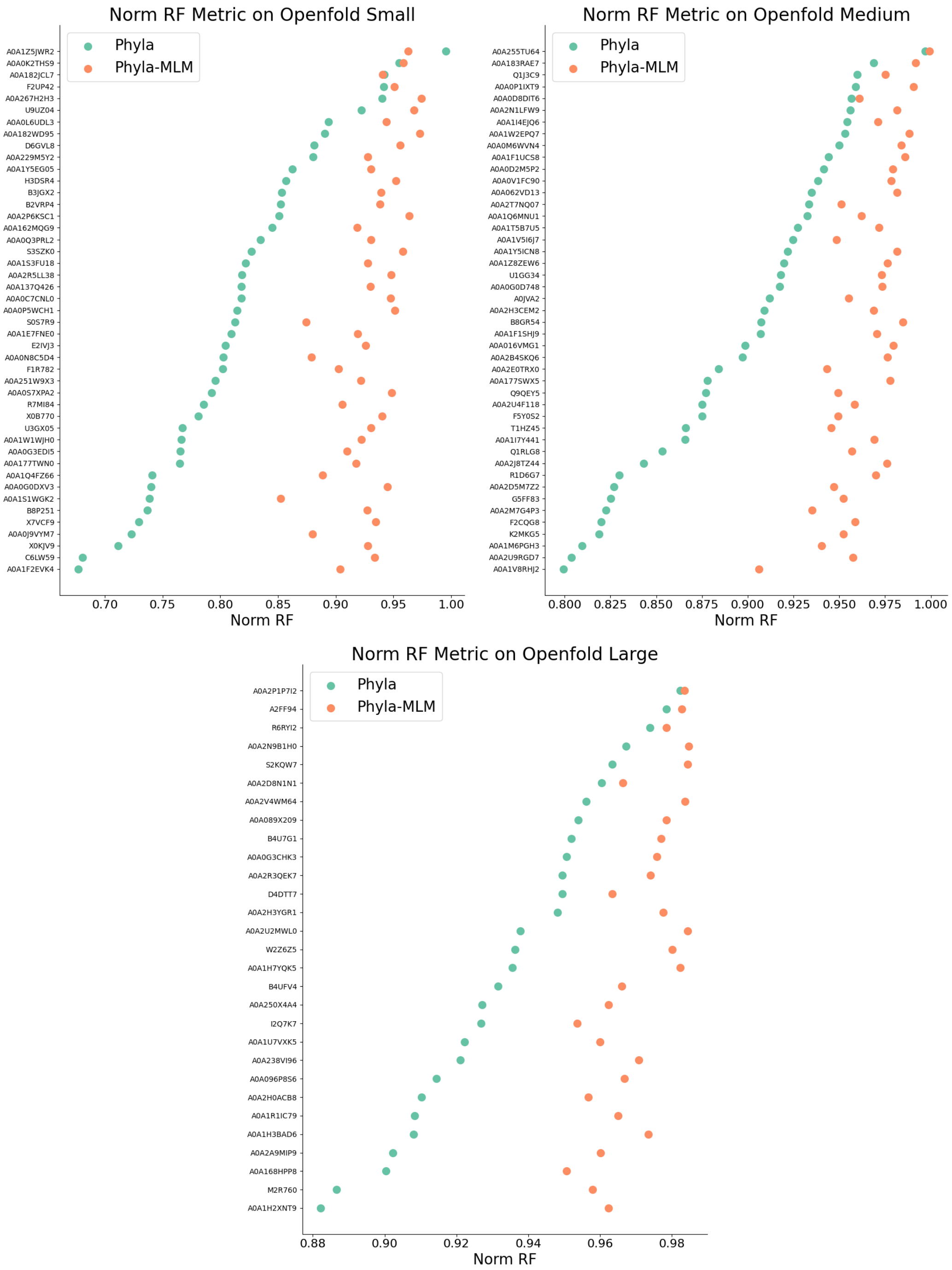

Figure S7: (a) Norm RF metric (lower, i.e. to the left, is better) values for all datasets in the Openfold Small (0 to 1000 sequences) evaluation set for Phyla vs Phyla-MLM. (b) Norm RF metric (lower, i.e. to the left, is better) values for all datasets in the Openfold Medium (1000 to 2000 sequences) evaluation set for Phyla vs Phyla-MLM. (c) Norm RF metric (lower, i.e. to the left, is better) values for all datasets in the Openfold Large (2000+ sequences) evaluation set for Phyla vs Phyla-MLM.

bioRxiv preprint doi: https://doi.org/10.1101/2025.01.17.633626; this version posted January 22, 2025. The copyright holder for this preprint (which was not certified by peer review) is the author/funder, who has granted bioRxiv a license to display the preprint in perpetuity. It is made available under aCC-BY 4.0 International license.

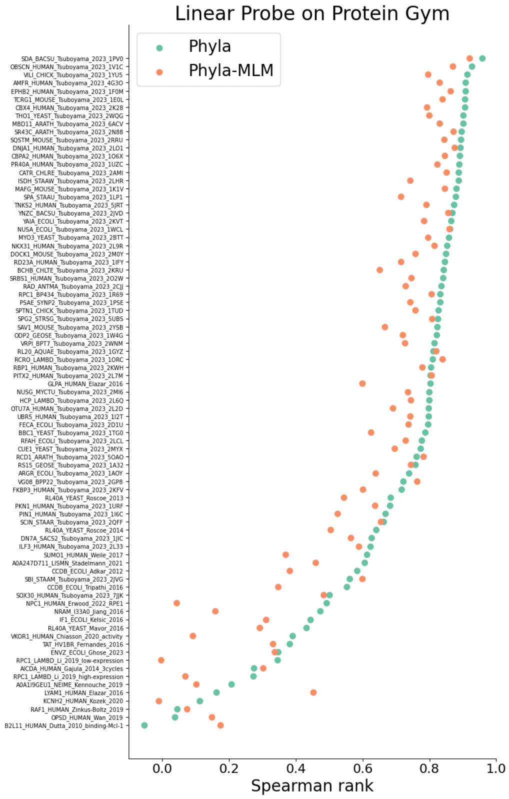

Figure S8: (a) Spearman correlation metric (higher, i.e. to the right, is better) using linear probe calculation method values for 83 datasets in the Protein Gym evaluation set for Phyla vs Phyla-MLM.
